# Microbial Taxa Distribution Is Associated with Ecological Trophic Cascades along an Elevation Gradient

**DOI:** 10.3389/fmicb.2017.02071

**Published:** 2017-10-27

**Authors:** Fei Yao, Shan Yang, Zhirui Wang, Xue Wang, Ji Ye, Xugao Wang, Jennifer M. DeBruyn, Xue Feng, Yong Jiang, Hui Li

**Affiliations:** ^1^CAS Key Laboratory of Forest Ecology and Management, Institute of Applied Ecology, Chinese Academy of Sciences, Shenyang, China; ^2^University of Chinese Academy of Sciences, Beijing, China; ^3^College of Land and Environment, Shenyang Agricultural University, Shenyang, China; ^4^Department of Biosystems Engineering & Soil Science, University of Tennessee, Knoxville, TN, United States

**Keywords:** microbial biogeography, soil bacteria, soil fungi, amplicon sequencing, microbial taxonomic survey, oligotrophic-copiotrophic theory, elevational gradient

## Abstract

The elevational pattern of soil microbial diversity along mountain slopes has received considerable interest over the last decade. An increasing amount of taxonomic data on soil microbial community composition along elevation gradients have been collected, however the trophic patterns and environmental drivers of elevational changes remain largely unclear. Here, we examined the distribution patterns of major soil bacterial and fungal taxa along the northern slope of Changbai Mountain, Northeast China, at five typical vegetation types located between 740 and 2,691 m above sea level. Elevational patterns of the relative abundance of specific microbial taxa could be partially explained by the oligotrophic-copiotrophic theory. Specifically, two dark-coniferous forests, located at mid-elevation sites, were considered to be oligotrophic habitats, with relatively higher soil C/N ratio and ${\text{NH}}_{4}^{+}$-N concentrations. As expected, oligotrophic microbial taxa, belonging to the bacterial phyla Acidobacteria and Gemmatimonadetes, and fungal phylum Basidiomycota, were predominant in the two dark-coniferous forests, exhibiting a mid-elevation maximum pattern. In contrast, the broad leaf-Korean pine mixed forest located at the foot of the mountain, *Betula ermanii*-dominated forest located below the tree line, and alpine tundra at the highest elevation were considered more copiotrophic habitats, characterized by higher substrate-induced-respiration rates and ${\text{NO}}_{3}^{-}$-N concentrations. Microbial taxa considered to be so called copiotrophic members, such as bacterial phyla Proteobacteria and Actinobacteria, and fungal phylum Ascomycota, were relatively abundant in these locations, resulting in a mid-elevation minimum pattern. At finer taxonomic levels, the two most abundant proteobacterial classes, alpha- and beta-Proteobacteria, along with Acidobacteria Gp1, 2, 3, 15, and the Basidiomycotal class of Tremellomycetes were classified with the copiotrophic group. Gamma- and delta-Proteobacteria, Acidobacteria Gp4, 6, 7, 16, and Basidiomycotal class of Agaricomycetes were classified as oligotrophic taxa. This work uses the oligotrophic-copiotrophic theory to explain the elevational distribution pattern of the relative abundance of specific microbial taxa, confirming some of the existing trophic classifications of microbial taxa and expanding on the theory to include a broader range of taxonomic levels.

## Introduction

The oligotrophic-copiotrophic theory has been proposed as an ecological classification scheme for soil bacteria, predicting that copiotrophic taxa are more associated with greater labile carbon (C) pools and flourish in soils with higher net carbon mineralization rate, whereas the oligotrophic taxa dominate soils with lower organic carbon availability (Fierer et al., 2007). The soil carbon/nitrogen (C/N) ratio is also proposed as an important indicator of C availability (Wichern et al., 2007; Muller et al., 2017), with high C/N ratio representing low C availability. According to this theory, a variety of dominant soil bacterial phyla, such as Proteobacteria (in particular Alpha- and Beta-Proteobacteria) (Fierer et al., 2007; Francioli et al., 2016) and Firmicutes (Cleveland et al., 2007; Nemergut et al., 2010; Francioli et al., 2016) have been generally classified as copiotrophic microbes, whereas Acidobacteria (Smit et al., 2001; Fierer et al., 2012), Gemmatimonadetes (Cederlund et al., 2014), Verrucomicrobia (Janssen et al., 1997, 2002; Bergmann et al., 2011; Cederlund et al., 2014) and Chloroflexi (Phung et al., 2004; Ramirez-Villanueva et al., 2015) are typically classified as oligotrophic microbes. The trophic categorization of Actinobacteria has remained unclear thus far. Previous studies examining the response of Actinobacteria to nutrient additions have documented mixed results, with some studies reporting an increase in relative abundance (Ramirez et al., 2010, 2012; Pan et al., 2014), while others reported no change (Fierer et al., 2007; Zhao et al., 2014; Li et al., 2016).

Though the oligotrophic-copiotrophic theory has often been used to discuss the ecological roles of dominant bacterial phyla, it is less frequently applied to fungal taxa. Saprotrophic fungi are known to prefer nutrient-rich environments (Colpaert and vanTichelen, 1996) and play important roles in wood and leaf-litter decomposition and nutrient cycling (Crowther et al., 2012; Kohler et al., 2015). In contrast, ectomycorrhizal (ECM) fungi usually grow under oligotrophic conditions, such as the deeper soil horizons (Lindahl et al., 2007). They typically have a slow growth rate (Hibbett et al., 2000), and have a limited capacity to affect the decomposition of organic materials (Kohler et al., 2015). Based on these previous findings, we proposed that ECM fungal taxa may be characterized as oligotrophic, while saprotrophic fungi may exhibit copiotrophic tendencies.

The distribution patterns of organisms and biological communities along elevational gradients is a fundamental subject in biogeography (Linnaeus, 1781; Bryant et al., 2008), because elevational gradients are characterized by distinct climate and ecological changes over short geographic distances. Studies have documented elevational patterns of diversity across a wide variety of taxonomic groups, such as trees, mammals, birds, insects, and nematodes (Bryant et al., 2008; Ohsawa and Ide, 2008; Pyrcz et al., 2009; Rowe, 2009; Zhang et al., 2012). With the application of molecular methods in microbial ecology, the elevational distribution patterns of soil microbial communities has received increasing attention over the last decade (Bryant et al., 2008; Fierer et al., 2011). A number of studies have documented decreasing alpha-diversity with elevation (Bryant et al., 2008; Bahram et al., 2012; Zhang et al., 2015), whereas others reported a maximum diversity at mid-elevations (Singh et al., 2012), or non-significant elevational patterns (Fierer et al., 2011; Roy et al., 2013; Shen et al., 2013, 2014; Singh et al., 2013; Geml et al., 2014; Wang J. T. et al., 2015). Since it is known that individual microbial taxa exhibit different life strategies, for example, Proteobacteria are usually active in metabolizing labile carbon (Fierer et al., 2007), while Acidobacteria tend to be associated with habitats with more recalcitrant carbon (Llado et al., 2016), determining the effects of elevational gradients on the relative abundances of specific microbial taxa maybe important for interpreting patterns of microbial diversity and understanding ecosystem functioning.

The elevational distribution patterns of the relative abundance of specific soil microbial taxa had garnered less attention, and thus mechanisms driving taxonomic distribution patterns are poorly understood. A study conducted in Mount Fuji, Japan demonstrated that Proteobacteria and Acidobacteria decreased in relative abundance with increasing elevation, whereas Actinobacteria, Chloroflexi, and Gemmatimonadetes showed a reverse trend (Singh et al., 2012). On Shennongjia Mountain in central China, which has the world's most well-preserved subtropical virgin and mature forest in a mid-latitudinal area, relative abundances of Acidobacteria, Actinobacteria, and Alphaproteobacteria was lowest at the middle elevations, while Verrucomicrobia and Betaproteobacteria exhibited the highest relative abundances at mid-elevation (Zhang et al., 2015). Observations from another subtropical forest in China (Lushan Mountain) also revealed that at mid elevations the relative abundance of Acidobacteria and Ascomycota was lowest and Proteobacteria and Basidiomycota were highest (Meng et al., 2013). Leotiomycetes, a dominant class within the Ascomycota, increased in relative abundance with elevation, exhibiting their highest relative abundance at high elevation in a tropical forest in the Peruvian Andes (Meier et al., 2010). Despite the increasing amount of descriptive data, mechanisms driving these distinct elevational patterns are not clearly discussed. It has been proposed that low pH could be an important factor associated with the prevalence of Acidobacteria in specific habitats (Singh et al., 2012). Moreover, the higher soil total carbon and C/N ratio have been suggested to be closely associated with increasing Basidiomycota, and diminishing Ascomycota abundances (Meng et al., 2013).

The Changbai Mountain Natural Reserve, located along the border of China and North Korea, is one of the best preserved natural ecosystems in Northeast China. The vertical distribution of vegetation along the northern slope of Changbai Mt. mirrors the latitudinal vegetation gradient from temperate to polar zones on the Eurasian continent (Xu et al., 2004; Zhang et al., 2011), and thus presents an ideal site for investigating elevational distribution patterns of soil microbes. Five typical vegetation types, including broad leaf-Korean pine mixed forest (BL-KP) (740–1,100 m), Korean pine-spruce fir forest (KP-SF) (1,100–1,500 m), Spruce Fir-*Betula ermanii* forest (SF-BE) (1,500–1,800 m), *Betula ermanii* forest (BE) (1,800–2,100 m) and Alpine tundra (AT) (2,100–2,691 m) were chosen for this study to elucidate the distribution patterns of dominant soil microbial taxa abundance along the northern slope of Changbai Mountain. We hypothesized that different vegetation types along the elevational gradients would select distinct soil bacterial and fungal communities, with copiotrophic taxa predominating in nutrient-rich forests and the predicted oligotrophic microbial taxa flourishing in nutrient-poor habitats. Specifically, we hypothesized that the two dark-coniferous forest sites (KP-SF and SF-BE) at mid-elevation would select for oligotrophic taxa because of the high C/N ratio and lignin in needle litters (Knorr et al., 2005; Zhang et al., 2016), and thus, the low C availability in soil. As a result, these coniferous forests might be dominated by proposed oligotrophic microbial taxa. In contrast, forests with more broad leaf trees, including BL-KP and BE, would have higher proportions of copiotrophic microbial taxa. At the highest elevation, alpine tundra is typically considered oligotrophic because of harsh environmental conditions, including high solar radiation, low humidity, large daily temperature fluctuations, high wind exposure, and low soil nutrients (Freeman et al., 2009; King et al., 2012), and thus we predicted that the oligotrophic taxa would be predominant at the AT site.

The main objectives of our study were to: (1) document the taxonomic distributions of soil bacteria and fungi along an elevation gradient; (2) determine if distributions follow patterns predicted by the oligotrophic-copiotrophic theory; (3) correlate distribution patterns to edaphic factors which may help explain the ecological trophic cascades along an elevation gradient.

## Materials and methods

### Site description

The experimental site is located in the Changbai Mountain Natural Reserve (41°23′-42°36′N, 126°55′-129°00′E), Jilin Province, Northeast China. The Reserve has been protected from logging and other anthropogenic disturbances since it was established in 1960, and joined the World Biosphere Reserve Network under the UNESCO Man and the Biosphere Program in 1980. This region has a typical temperate continental monsoon climate. The northern slope of Changbai Mountain has a moderate slope (<3%). With increasing altitude from 740 to 2,691 m above sea level, annual mean temperature changes from 3 to −7°C, and annual mean precipitation increases from 759 to 1,340 mm (He et al., 2005). The climatic variations along the northern slope form a clear vertical pattern of vegetation types, mirroring the latitudinal vegetation gradient from temperate to polar zones on the Eurasian continent.

Five typical vegetation types along the elevational gradient on the northern slope of Changbai Mt. were selected in this study: (i) Broad leaf-Korean pine mixed forest (BL-KP) (42°23′, 128°05′) located between 740 and 1,100 m was dominated by a mixture of Korean pine (*Pinus koraiensis*) and broad leaf trees, including aspen (*Populus davidiana Dode*), birch (*Betula platyphylla Suk*), basswood (*Tilia amurensis Rupr*), oak (*Quercus mongolica*), maple (*Acer mono Maxim*), and elm (*Ulmus pumila L*.). The annual mean temperature (AMT) was 2.6°C, and the annual mean precipitation (AMP) was 691 mm, with soils classified as Albi-Boric Argosols according to the US Soil Taxonomy classification. (ii) Korean pine-Spruce fir forest community (KP-SF) (42°08′, 128°07′) located at 1,100–1,500 m was dominated by spruce (*Picea jezoensis*), fir (*Abies nephrolepis*), and Korean pine (*Pinus koraiensis*). The AMT was 0.3°C, and the AMP was 811 mm with soils classified as Bori-Udic Cambosols. (iii) Spruce fir-*Betula ermanii* forest community (SF-BE) ranged from 1,500 to 1,800 m (42°04′, 128°03′), and the dominant tree species were spruce (*Picea jezoensis*) and fir (*Abies nephrolepis*), mixed with a few stands of *Betula ermanii*. The AMT was −2.3°C, and the AMP was 967 mm. Soil was classified as Umbri-Gelic Cambosols. (iv) *Betula ermanii* forest (BE) (42°03′, 128°04′) was located between 1,800 and 2,100 m, and was dominated by *Betula ermanii*. The AMT was −3.3°C, and the AMP was 1,038 mm. Soils were classified as Permi-Gelic Cambosols. (v) Alpine tundra (AT) (42°02′, 128°03′) was located between 2,100 and 2,691 m, and the dominant vegetation was *Vaccinium vitis-idaea, Rhododendronaureum, Carex dispalata*, and *Dryas octopetala*. The AMT was −4.8°C, and the AMP was 1,154 mm. The soil was classified as Permafrost cold Cambisols (Xu et al., 2004; He et al., 2005; Shen et al., 2014).

### Soil sample collection and analysis

Soil samples were collected from six replicate plots (20 × 20 m) adjacent to each other at each of the five vegetation types in early September 2014. To avoid the edge effect, all the soil samples were collected from the center of each vegetation zone, at least 50 m away from the upper and the lower boundary. The six replicate plots were evenly distributed within the center zone of each forest type. At each of the six experimental plots, five soil cores were randomly collected and mixed together to form a composite sample for the plot. Top soil (0–10 cm) of each soil core was collected using a 5 cm diameter PVC core soil sampler after the litter layer had been removed. Visible roots and residues were removed prior to homogenizing the soil cores. The composite samples were sieved at a mesh size of 2 mm, and transported to the laboratory on ice within 48 h of sampling. The pooled soil samples were subdivided into three subsamples. The subsamples for determining soil physicochemical parameters were air-dried at ambient temperature until constant weight. For biological parameters, such as biomass and respiration, soils were stored at 4°C, and subsamples for molecular analysis were kept at −80°C.

The following soil characteristics were determined for each sample and used in the subsequent analyses: total carbon (TC), total nitrogen (TN), total sulfur (TS), C/N ratio, nitrate-N (${\text{NO}}_{3}^{-}$-N), ammonium-N (${\text{NH}}_{4}^{+}$-N), gravimetric moisture content, pH, microbial biomass carbon (MBC), microbial biomass nitrogen (MBN), basal respiration (BR), substrate induced respiration (SIR), and carbon availability index (CAI). Soil TC, TN, and TS were quantified using Elemental Analyzer (2400II CHN elemental analyzer; Perkin-Elmer, USA) after grinding by a Brinkmann Retsch mill (Haan, Germany). Soil C/N ratio was calculated using TC and TN dataset. ${\text{NO}}_{3}^{-}$-N and ${\text{NH}}_{4}^{+}$-N were extracted in 2 M KCl, and measured using a continuous-flow ion auto-analyzer (Scalar SANplus segmented flow analyzer, The Netherlands). Gravimetric soil moisture content was determined by drying overnight at 105°C and re-weighing to measure water loss. Soil pH was measured from a soil/water (1:2.5) suspension using PHS-3G digital pH meter (Precision and Scientific Crop, Shanghai, China). Soil MBC and MBN was measured using a chloroform fumigation extraction method (Brookes et al., 1985). Basal soil respiration (BR) was measured on soil samples that were incubated for 24 h in a closed vessel; headspace CO_2_ was measured using a Li-COR 8200 Infrared Gas Analyzer (IRGA) (Li-COR Biosciences, Lincoln, NB, USA). SIR was assayed as CO_2_ evolution within 2 h after glucose addition. CAI was calculated by dividing BR by SIR (Gershenson et al., 2009).

### DNA extraction, amplification and sequencing

Soil genomic DNA was extracted from 0.25 g of moist soil samples using a MoBio PowerSoil® DNA Isolation extraction kit (MoBio Laboratories Inc. Carlsbad, California, USA) following the manufacturer's instructions. The extracted DNA was quantified with a Nanodrop UV-Vis spectrophotometer (NanoDrop products, Wilmington, DE). The V_1_-V_3_ region of the bacterial 16S rRNA gene was amplified using the primers of 27F (5′-AGAGTTTGATCCTGGCTCAG-3′) and 533R (5′-TTACCGCGGCTGCTGGCAC-3′) (Weisburg et al., 1991). The primers 1737F (5′-GGAAGTAAAAGTCGTAACAAGG-3′) and 2043R (5′-GCTGCGTTCTTCATC GATGC-3′) were used to amplify the fungal ITS region (Bellemain et al., 2010). Both forward and reverse primers were tagged with adapter, pad, linker, and 6 bp-barcode sequences. The 16S rRNA PCR reaction and amplification program were performed according to the protocols described in Li et al. (2016). Amplification of fungal ITS region was performed in triplicate in 20 μl mixtures which contained 4 μl of 5 × FastPfu Buffer, 2 μl of 2.5 mM dNTPs, 0.8 μl of each primer (5 μM), 0.4 μl of FastPfu Polymerase, 0.2 μl of BSA and 10 ng of template DNA. The thermocycler conditions were: 35 cycles of 95°C for 30 s, 55°C for 30 s and 72°C for 45 s, followed by a final extension at 72°C for 10 min. The amplified 16S rRNA gene and ITS region were sequenced using 300PE (paired-end) and 250 PE reads respectively, on the Illumina Miseq platform (Illumina, USA) at Majorbio Bio-Pharm Technology Co., Ltd., Shanghai, China.

Raw sequences files were demultiplexed, trimmed of reads containing ambiguous bases and long homopolymers, and merged using QIIME v 1.7.0 (Caporaso et al., 2012). Unique sequences were sorted by abundance, and singletons in the data set were discarded. All quality-filtered sequences from 16S rRNA and ITS gene amplicons were clustered into OTUs (Operational Taxonomic Unit) at 97% similarity cutoff using UPARSE (version 7.1, http://drive5.com/uparse/), followed by chimera filtering using the Ribosomal Database Project (RDP) (Wang et al., 2007) and UCHIME (Edgar et al., 2011). To normalize all libraries to the same size, a subset of 8,000 sequences and 10,486 sequences per sample were randomly selected from bacterial 16S rRNA and fungal ITS datasets, respectively. Representative prokaryotic and fungal OTU sequences were classified taxonomically using the RDP classifier against Greengenes and UNITE reference databases, respectively. The census at each taxonomical level was processed with Perl scripts. The raw sequence data have been deposited in NCBI SRA under the accession number SRP061305.

### Identification of ECM and saprotrophic fungi

The soil fungal community could be roughly divided into two ecological functional groups, represented by ECM and saprotrophic fungi (Leake et al., 2003; Read and Perez-Moreno, 2003; Kohler et al., 2015). We tried to identify genera that could be grouped either into ECM or into saprotrophic (or potential saprotrophic) fungi, based on previous published literature (Frankland, 1998; Tedersoo et al., 2010; Branco et al., 2013). ECM fungi generally fall within the phylum of Basidiomycota, and most of the saprotrophic fungi are phylogenetically classified as phylum Ascomycota (Table S1).

### Statistical analysis

*T*-test was used to assess the differences in soil properties and the relative abundance of dominant microbial taxa among different vegetation types. A Bonferroni correction was done for multiple comparisons (*P*-value was corrected based on the number of comparisons). Soil variables and the relative abundance of microbial taxa which did not follow a normal distribution were ln-transformed.

Bray-Curtis distances of microbial community composition were computed using the vegan package in R (http://www.r-project.org/) based on OTU tables, and were then visualized using non-metric multidimensional scaling (NMDS) plots as implemented in PRIMER v6 (PRIMER-E, Ltd.). To determine whether vegetation type significantly influenced microbial community composition, permutational multivariate analysis of variance was performed using the ADONIS function implemented in the R statistical environment (v 3.3.1), based on the Bray-Curtis dissimilarity matrix. To identify the relative importance of vegetation type and multiple soil variables contributing to the changes of microbial community composition, partial canonical correlation analysis (CCA) and subsequent Monte Carlo test (999 permutations) were performed using vegan package in R. Soil parameters used in CCA analysis were pre-selected by BIO-ENV function implemented in R vegan. Redundancy analysis (RDA) was performed with the software CANOCO 5.0 to further evaluate the relationships between soil microbial biomass, soil respiration parameters and selected microbial taxa.

Pearson's correlation was used to analyze the correlation between relative abundance of specific microbial taxa and soil properties by R “corrplot” package (version 3.3.1). Because of the non-independency of the replicates in this experimental design, a mixed model approach was used to analyze relationships between soil parameter and taxa relative abundances: Hierarchical Linear Models (HLM) were used to further explain the variation in the relative abundance of dominant microbial taxa using R “lme4” and “lmeTest” packages. Because of the collinearity among environmental factors, we pre-selected the parameters by removing variables with higher correlation (*r*^2^ > 0.6). Soil pH, SIR, moisture, ${\text{NO}}_{3}^{-}$-N, TN, BR, ${\text{NH}}_{4}^{+}$-N, C/N and CAI were selected as the individual-level variables in the HLM analysis. Vegetation type was considered the group-level variable. The best fitting models were selected based on the lowest AIC and BIC values.

## Results

### Soil physico-chemical properties along the elevational gradients

All soil physical and chemical properties measured in this study showed significant variations along the elevational gradient, except TC and TS (Table 1). The C/N ratios in BL-KP and BE forests, the two forests with more broad leaf trees, were much lower than the two dark-coniferous forests (KP-SF and SF-BE). Soil ${\text{NO}}_{3}^{-}$-N and ${\text{NH}}_{4}^{+}$-N showed the reverse pattern along the elevational gradient. Soil ${\text{NH}}_{4}^{+}$-N concentrations showed a mid-elevation maximum pattern, with higher values being observed in the two dark-coniferous forests. In contrast, soil ${\text{NO}}_{3}^{-}$-N concentrations was lowest at mid-elevation. The BL-KP forest located at the mountain foot and AT located at the summit contained more soil ${\text{NO}}_{3}^{-}$-N than the other vegetation types. The highest soil moisture value was observed at the lowest altitude (BL-KP forest). Soil pH varied significantly (*t*-test*, P*_*adjust*_ < 0.05) across different vegetation types, ranging from 4.81 to 5.36. In general, the pH values of BL-KP, BE, and AT were higher than that of the two dark-coniferous forests.

**Table 1:** Soil properties along the northern slope of Changbai Mt.

**Vegetation**	**Elevation**	TC^*^(g kg^−1^)	**TN (g kg^−1^)**	**TS (g kg^−1^)**	**C/N**	**${\text{NO}}_{3}^{-}$-N (mg kg^−1^)**	**${\text{NH}}_{4}^{+}$-N (mg kg^−1^)**	**Moisture (%)**	**pH**
BL-KP	740–110 m	117.50a^**^ (14.72)	9.56a (0.99)	1.18a (0.17)	12.22c (0.46)	9.86a (0.97)	28.38b (4.89)	0.36a (0.02)	5.36a (0.06)
KP-SF	1,100–1,500 m	101.95a (24.75)	4.59b (0.95)	0.83a (0.08)	21.52a (1.01)	4.60b (0.41)	30.27b (5.17)	0.25b (0.02)	4.92b (0.10)
SF-BE	1,500–1,800 m	100.12a (8.42)	6.51ab (0.37)	1.00a (0.06)	15.29b (0.45)	7.29ab (1.88)	72.88a (7.94)	0.28ab (0.02)	4.81b (0.04)
BE	1,800–2,100 m	65.700a (10.18)	5.29b (0.76)	0.7a (0.06)	12.38c (0.34)	6.20ab (0.73)	48.48ab (11.41)	0.3ab (0.01)	5.17ab (0.13)
AT	2,100–2,691 m	109.85a (12.32)	6.01ab (0.56)	0.84a (0.06)	18.27ab (0.97)	15.0ab (5.93)	28.79b (8.58)	0.3ab (0.02)	5.2ab (0.14)

*Shown are the mean values, and the values in parentheses are standard error (n = 6). BL-KP, Broad leaf-Korean pine mixed forest; KP-SF, Korean pine-spruce fir forest; SF-BE, Spruce Fir-Betula ermanii forest; BE, Betula ermanii forest; AT, Alpine tundra*.

* *TC, total carbon; TN, total nitrogen; TS, total sulfur; C/N, carbon:nitrogen ratio*.

** *Different letters indicate significant differences (t-test, P_adjust_ < 0.05) among vegetation types*.

**Figure 1:**
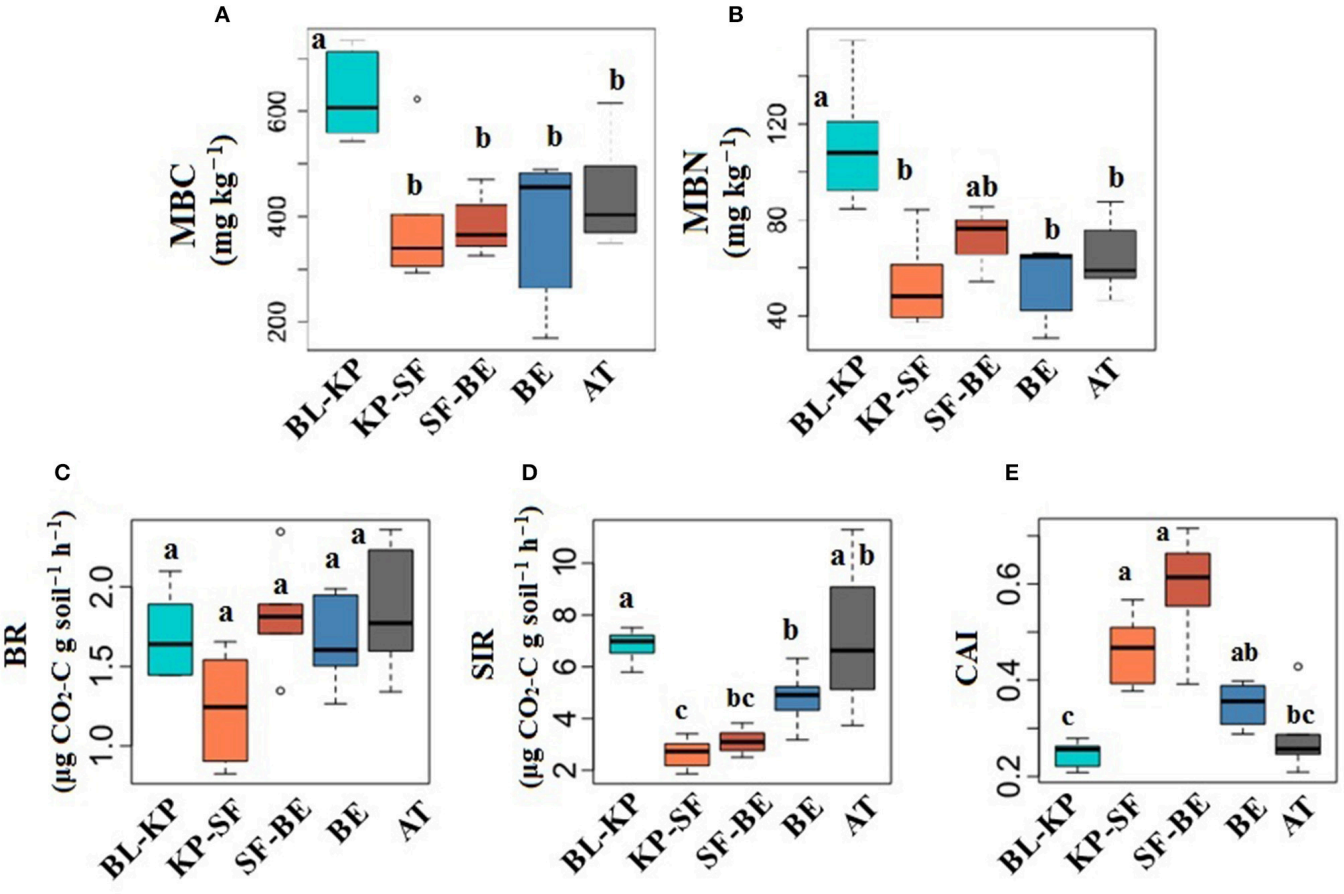
Changes in soil microbial biomass **(A)** microbial biomass carbon, MBC; **(B)** microbial biomass nitrogen, MBN; and soil respiration **(C)** basal respiration, BR; **(D)** substrate induced respiration, SIR; **(E)** carbon availability index, (CAI) under different vegetation types along an elevational gradient in Changbai Mountain. Different letters indicate significant differences (*t*-test, *P*_*adjust*_ < 0.05) between sites.

The highest MBC and MBN were both observed in BL-KP forest (Figures 1A,B), significant different with other forest types (*t*-test, *P*_*adjust*_ < 0.05). Soil basal respiration (BR) was lowest at the KP-SF site, indicating the potential lower activity of soil organisms in this coniferous forest (Figure 1C). SIR, a key indicator of the activities of living biomass in soils, showed was lowest at mid-elevation (Figure 1D): The BL-KP, BE forest and AT exhibited higher SIR than the two dark coniferous forests. The carbon availability index (CAI), calculated by dividing the basal respiration rate by SIR rate, was significantly different (*t*-test, *P*_*adjust*_ < 0.05) in the two dark-coniferous forest (ranging from 0.46 to 0.59) compared to the other locations (ranging from 0.25 to 0.35) (Figure 1E), largely driven by a decrease in SIR.

### Variations in the overall microbial community composition across different vegetation types

**Figure 2:**
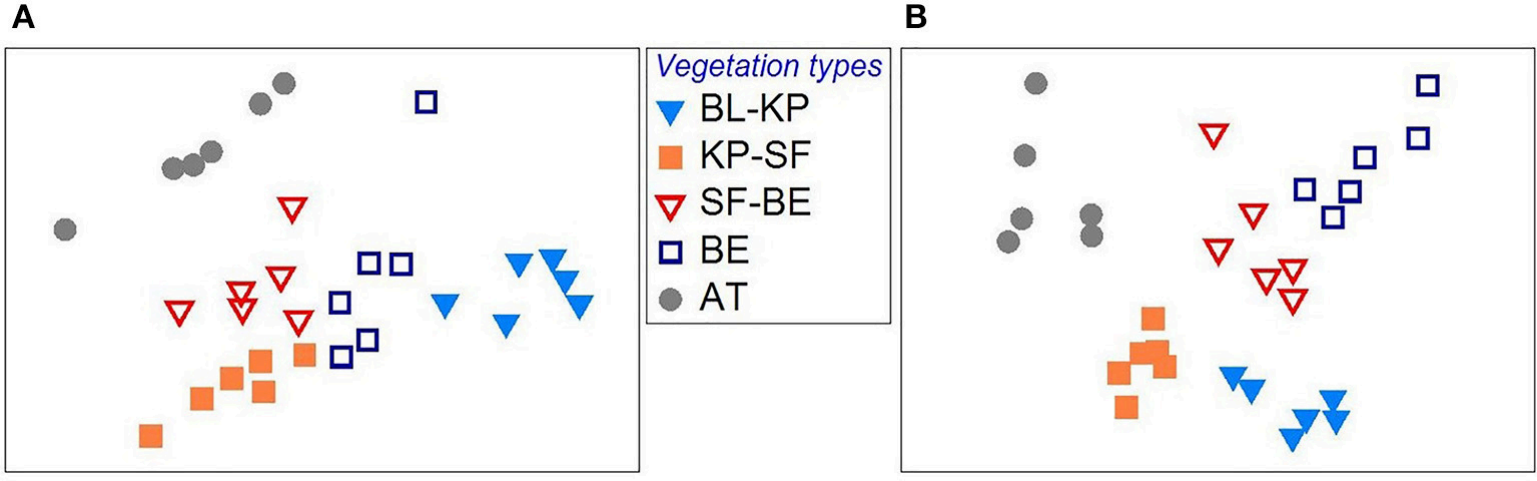
The variation in bacterial **(A)** and fungal **(B)** community composition across the elevational gradient on Changbai Mt., as revealed by Non Metric Multidimensional Scaling (NMDS) plot based on Bray-Curtis distances. BL-KP, Broad leaf-Korean pine mixed forest; KP-SF, Korean pine-spruce fir forest; SF-BE, Spruce Fir-*Betula ermanii* forest; BE, *Betula ermanii* forest; AT, Alpine tundra.

It was observed that the overall microbial community composition was significantly separated among different vegetation types, with respect to both bacterial (Figure 2A) and fungal (Figure 2B) communities (ADONIS, *P* < 0.01 for both cases), indicating the significant contribution of vegetation type in shaping the soil microbial communities along this elevation transect. The pair-wise ADONIS analysis also revealed significant separation of microbial community composition between any of the two vegetation types (Table S2).

Partial CCA was used to identify the relative importance of vegetation types and soil parameters contributing to the variations in microbial community composition (Table 2). The largest proportion of the variation in bacterial community composition was explained by vegetation type (15.13%, *P* < 0.05), followed by soil pH (3.78%), ${\text{NO}}_{3}^{-}$-N (3.26%), and C/N ratio (2.85%) (*P* < 0.05 for all soil variables). The community compositions of select bacterial phyla, Proteobacteria, Acidobacteria, Actinobacteria and Gemmatimonadetes, were also influenced significantly (*P* < 0.05 for all cases) by the vegetation type. However, the composition of each of these dominant bacterial phyla was influenced by different soil parameters (Table 2). The total fungal community composition was only significantly influenced by vegetation type (16.65%, *P* < 0.05). Phylum Basidiomycota was also influence by vegetation type (16.40%, *P* < 0.05), inferring the close relationship between this fungal population and the aboveground plants. Phylum Ascomycota, was significantly affected by both vegetation type (19.25%, *P* < 0.05) and multiple soil variables, including soil C/N ratio, ${\text{NO}}_{3}^{-}$-N concentrations and pH.

**Table 2:** Partial CCA (canonical correspondence analysis) of the relationship between vegetation types and BIO-ENV selected soil parameters and microbial community composition.

		**Vegetation types**	**${\text{NO}}_{3}^{-}$-N**	**C/N**	**MBN**	**SIR**	**CAI**	**pH**
**Bacteria**	Contribution	15.13%	3.26%	2.85%	2.11%	2.24%	2.00%	3.78%
	F	1.920	1.666	1.446	1.070	1.136	1.017	1.920
	*P*	**0.001**	**0.007**	**0.034**	0.345	0.245	0.455	**0.003**
Proteobacteria	Contribution	15.85%	3.56%	3.04%	1.86%	2.31%	2.10%	3.29%
	F	2.316	2.080	1.777	1.086	1.348	1.226	1.920
	*P*	**0.001**	**0.002**	**0.013**	0.341	**0.088**	0.173	**0.008**
Actinobacteria	Contribution	14.71%	2.54%	2.48%	1.927	1.64%	1.72%	4.80%
	F	2.088	1.430	1.417	1.094	0.931	0.975	2.726
	*P*	**0.001**	0.11	0.091	0.283	0.467	0.443	**0.004**
Acidobacteria	Contribution	15.8%	2.63%	2.54%	2.05%	1.85%	1.72%	3.73%
	F	2.291	1.524	1.473	1.186	1.074	0.996	2.165
	*P*	**0.001**	**0.022**	**0.044**	0.21	0.341	0.446	**0.001**
Gemmatimonadetes	Contribution	17.50%	3.86%	2.91%	2.94%	2.84%	2.15%	4.61%
	F	1.874	1.654	1.247	1.259	1.217	0.922	1.980
	*P*	**0.001**	**0.018**	0.141	0.121	0.177	0.568	**0.006**
**Fungi**	Contribution	16.65%	3.89%	3.41%	2.81%	3.61%	3.62%	3.32%
	F	1.425	1.332	1.167	0.961	1.234	1.240	1.135
	*P*	**0.001**	0.065	0.231	0.553	0.158	0.142	0.28
Basidiomycota	Contribution	16.4%	3.71%	3.23%	3.17%	3.12%	3.58%	3.28%
	F	1.344	1.212	1.057	1.038	1.021	1.171	1.074
	*P*	**0.002**	0.17	0.428	0.433	0.468	0.237	0.375
Ascomycota	Contribution	19.25%	5.18%	4.83%	2.68%	4.02%	2.56%	3.69%
	F	2.074	2.233	2.080	1.156	1.734	1.103	1.589
	*P*	**0.001**	**0.003**	**0.014**	0.332	**0.027**	0.398	**0.036**

*Bold values indicate that the correlations are significant (P < 0.05, Monte Carlo test of significance)*.

### The relative abundances of dominant bacterial and fungal phyla along elevational gradients

**Figure 3:**
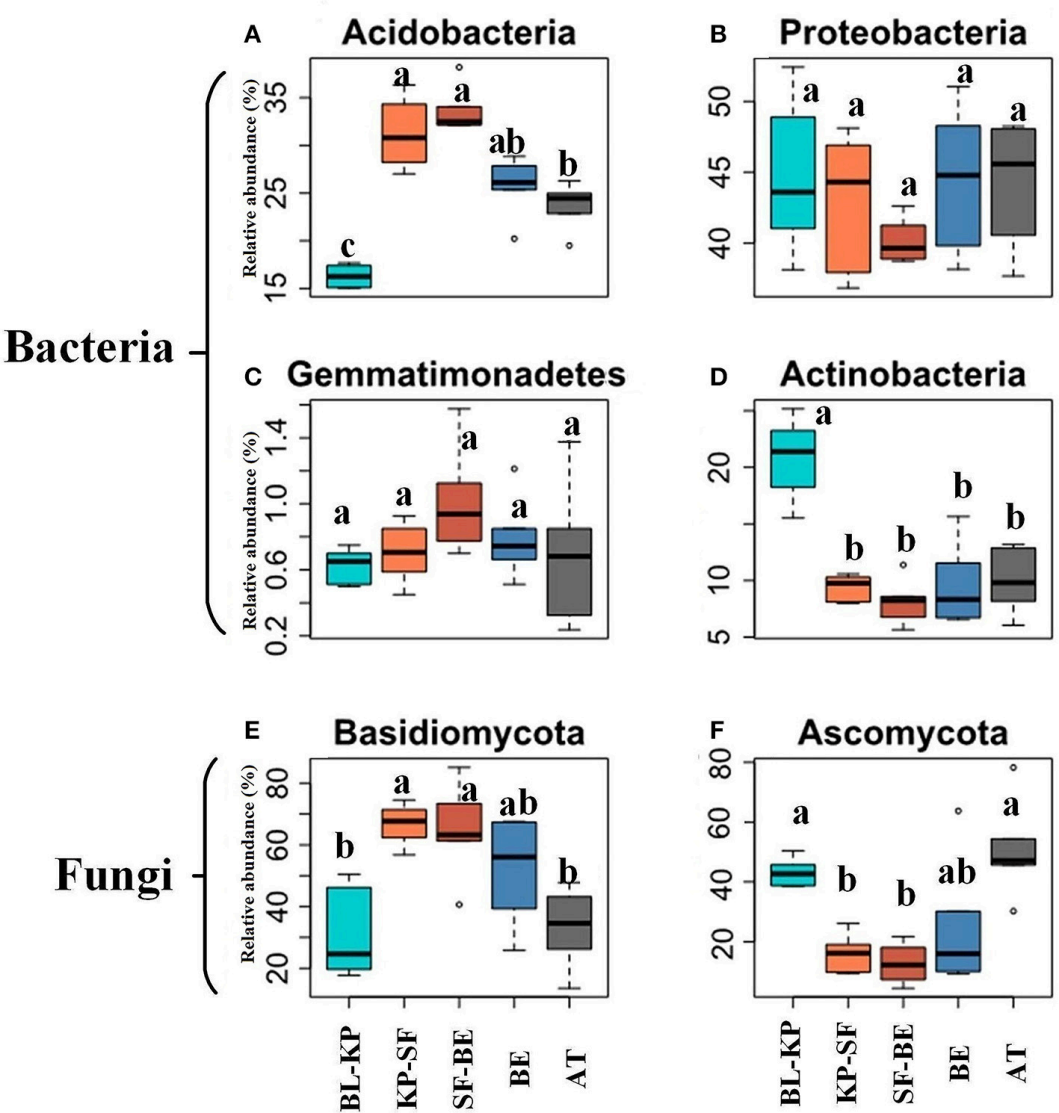
The relative abundances of selected bacterial and fungal phyla along the elevational gradients. The bacterial phyla of Acidobacteria **(A)** and Gemmatimonadetes **(C)**, and fungal phylum of Basidiomycota **(E)** demonstrated highest relative abundances at mid-elevation, whereas the bacterial phyla of Proteobacteria **(B)** and Actinobacteria **(D)**, and fungal phylum of Ascomycota **(F)** showed lowest relative abundances at mid elevation (right panel). Significant differences (*t*-test, *P*_*adjust*_ < 0.05) among vegetation types are marked by different letters. BL-KP, Broad leaf-Korean pine mixed forest; KP-SF, Korean pine-spruce fir forest; SF-BE, Spruce Fir-*Betula ermanii* forest; BE, *Betula ermanii* forest; AT, Alpine tundra.

The dominant phyla accounting for more than 0.5% relative abundance in the overall bacterial communities were Proteobacteria (mean relative abundance across all sites of 43.31%), Acidobacteria (26.12%), Actinobacteria (11.58%), Planctomycetes (4.05%), Verrucomicrobia (2.86%), Bacteroidetes (2.70%), Chloroflexi (2.37%), Firmicutes (0.85%) and Gemmatimonadetes (0.76%) (Figure S1A). The relative abundances of Acidobacteria (Figure 3A) is significantly greater in the two dark-coniferous forests. The relative abundance of Actinobacteria (Figure 3D) was greatest in BL-KP forest and lowest in SF-BE forest, showing a roughly mid-elevational minimum distribution. Although no significant difference in the relative abundance of Proteobacteria was detected across different elevations, the lowest relative abundances were observed at mid-elevations (Figure 3B).

Basidiomycota and Ascomycota were the two most abundant phyla detected in fungal communities, accounting for 50.69% and 29.08% of the total fungal sequences, respectively (Figure S1B). Interestingly, these two phyla showed reverse distribution patterns along the elevational gradients. The relative abundances of Basidiomycota were greater in the two dark-coniferous forests (Figure 3E), whereas the relative abundance of Ascomycota (Figure 3F) was significantly greater at the lowest and highest elevation sites (BL-KP and AT).

### The relative abundance of specific microbial classes along elevational gradients

**Figure 4:**
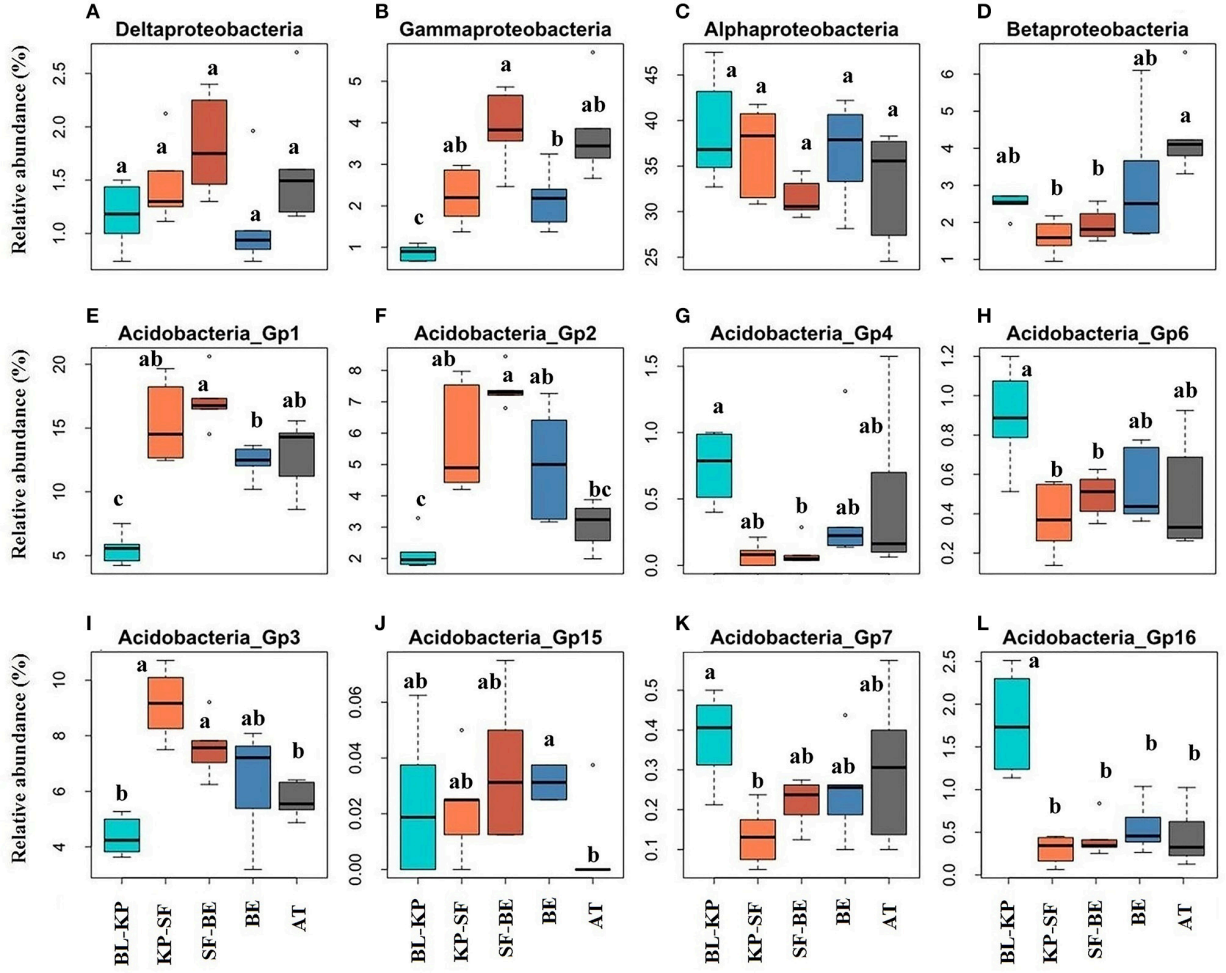
The elevational distribution patterns of the relative abundances of specific bacterial classes within the phylum of Acidobacteria and Proteobacteria. The classes within Proteobacteria are Deltaproteobacteria **(A)**, Gammaproteobacteria **(B)**, Alphaproteobacteria **(C)** and Betaproteobacteria **(D)**. Acidobacteria_Gp1 **(E)**, Acidobacteria_Gp2 **(F)**, Acidobacteria_Gp4 **(G)**, Acidobacteria_Gp6 **(H)**, Acidobacteria_Gp3 **(I)**, Acidobacteria_Gp15 **(J)**, Acidobacteria_Gp7 **(K)** and Acidobacteria_Gp16 **(L)** belong to the phylum of Acidobacteria. Significantly different (*t*-test, *P*_*adjust*_ < 0.05) groups are denoted with different letters. BL-KP, Broad leaf-Korean pine mixed forest; KP-SF, Korean pine-spruce fir forest; SF-BE, Spruce Fir-*Betula ermanii* forest; BE, *Betula ermanii* forest; AT, Alpine tundra.

The Alphaproteobacteria and Betaproteobacteria were the most two abundant Proteobacterial classes across all soil samples, accounting for 81.66 and 6.28% of all Proteobacteria sequences, respectively. The relative abundances of these two Proteobacterial classes were much lower in the two dark-coniferous forests (Figures 4C,D), consistent with the pattern for total Proteobacteria. In contrast, the less predominant classes Deltaproteobacteria and Gammaproteobacteria (represented 3.36 and 5.99% of all Proteobacterial sequences) generally exhibited an opposite pattern (Figures 4A,B), with higher proportions in the two dark-coniferous forests. AT also had similarly higher proportions of Gamma- and Delta-Proteobacteria to the dark coniferous sites.

In this experiment, 16 sub-groups of Acidobacteria were detected, including Gp1 (accounted for 46.73% of all Acidobacteria sequences), Gp2 (16.84%), Gp3 (25.27%), Gp4 (1.79%), Gp5 (0.54%), Gp6 (2.46%), Gp7 (1.14%), Gp10 (0.04%), Gp11 (0.15%), Gp12 (0.08%), Gp13 (0.08%), Gp15 (0.09%), Gp16 (3.48%), Gp17 (0.10%), Gp22 (0.005%), and Gp25 (0.02%). However, not all subgroups showed a similar distribution pattern along the elevational gradient. The relative abundances of subgroups Gp1, Gp2, Gp3, and Gp15 were much higher in the two dark-coniferous forests than the other vegetation types, showing a general mid-elevational maximum pattern (Figures 4E,F,I,J). In contrast, subgroups Gp4, Gp6, Gp7, and Gp16 were relatively more abundant in forests with broad leaf trees and AT, exhibiting a mid-elevational minimum pattern (Figures 4G,H,K,L).

**Figure 5:**
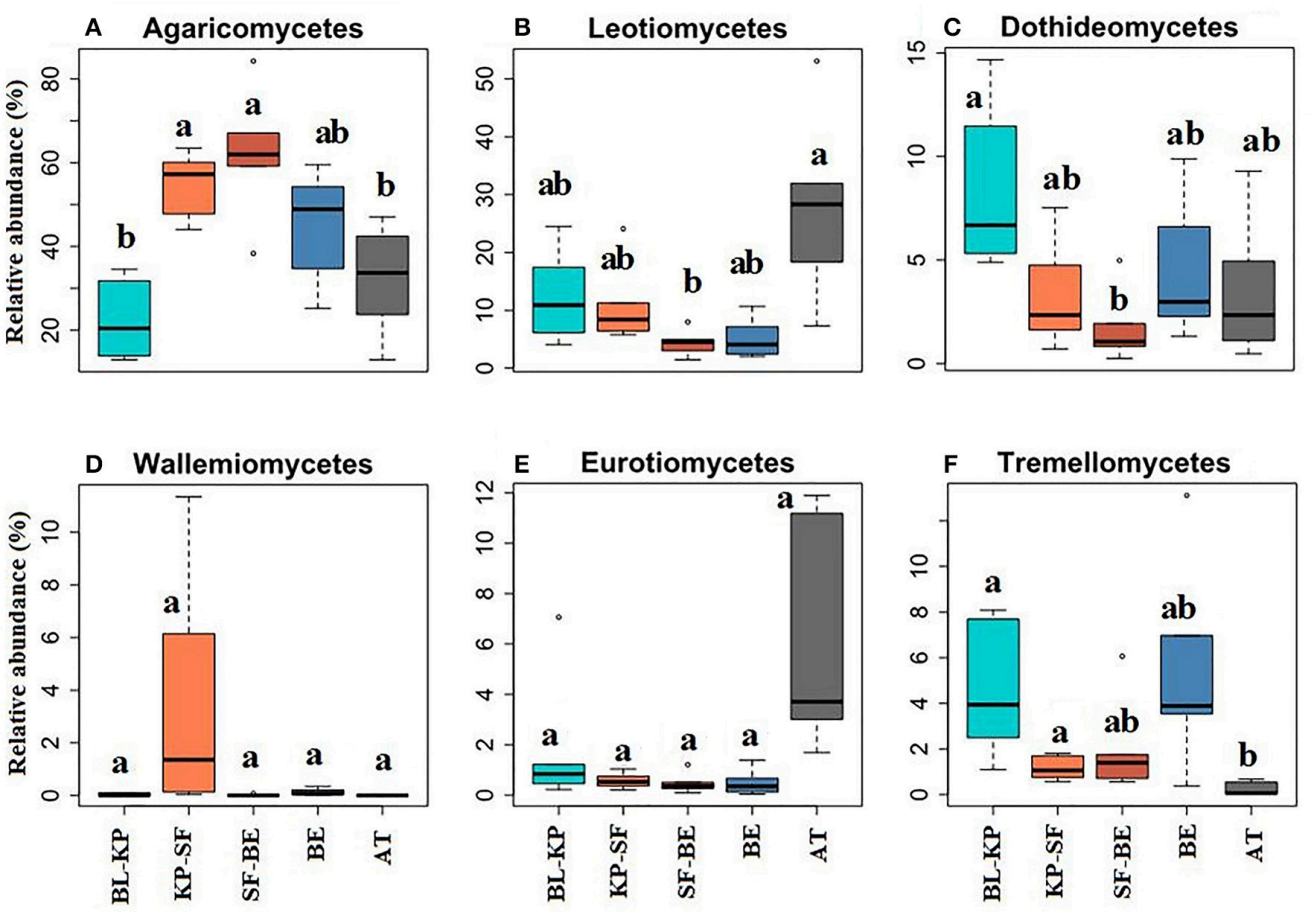
The elevational distribution patterns of the relative abundances of specific fungal classes within the phylum of Basidiomycota and Ascomycota. Agaricomycetes **(A)** and Wallemiomycetes **(D)** belong to Basidiomycota, Leotiomycetes **(B)**, Dothideomycetes **(C)**, Eurotiomycetes **(E)**, and Tremellomycetes **(F)** are classes within Ascomycota. Significantly different (*t*-test, *P*_*adjust*_ < 0.05) groups are denoted with different letters. BL-KP, Broad leaf-Korean pine mixed forest; KP-SF, Korean pine-spruce fir forest; SF-BE, Spruce Fir-*Betula ermanii* forest; BE, *Betula ermanii* forest; AT, Alpine tundra.

Basidiomycota and Ascomycota are the two predominant fungal phyla detected in this study, and more intensive analyses were conducted on these two fungal phyla to examine the patterns of specific fungal classes. We found that the most predominant class in Basidiomycota, Agaricomycetes (which accounted for 88.16% of the total Basidiomycotal sequences), followed the same elevational pattern (Figure 5A) with the phylum of Basidiomycota, with highest relative abundances at mid-elevation. Though it was not statistically significant, the class of Wallemiomycetes (1.05%) also showed a slight oligotrophic trend (Figure 5D), with the highest abundance being detected at KP-SF forest. However, another Basidiomycotal class, Tremellomycetes (5.97%), showed a reverse pattern (Figure 5F). The elevational distribution patterns of the Ascomycotal classes, Leotiomycetes (accounted for 42.51% of the total Ascomycotal sequences), Dothideomycetes (15.70%), and Eurotiomycetes (5.53%), were all in line with phylum Ascomycota, exhibiting a mid-elevational minimum pattern (Figures 5B,C,E). The Microbotryomycetes in Basidiomycota and the Sordariomycetes in Ascomycota both showed non-significant elevational patterns along the mountain slope.

### Distribution pattern of the relative abundances of ECM and saprotrophic fungi along the mountain slope

**Figure 6:**
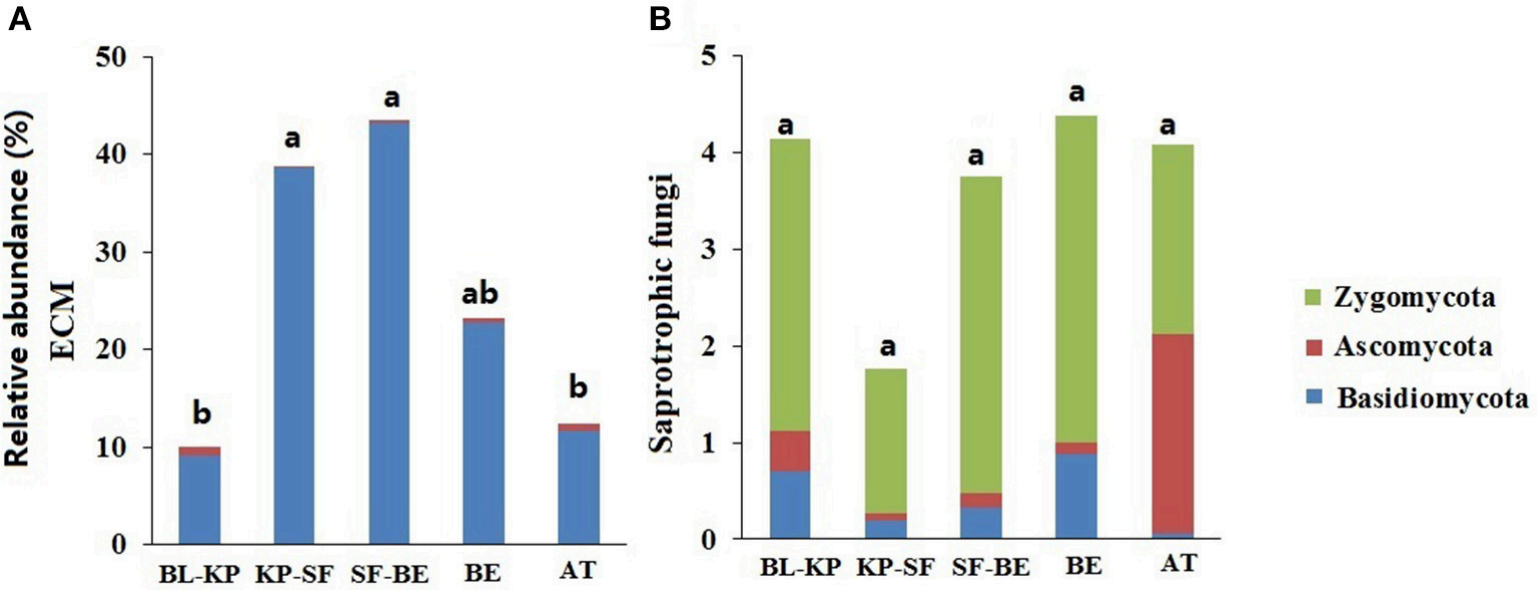
The elevational distribution patterns of the mean relative abundance of two fungal functional groups: ECM **(A)** and saprotrophic fungi **(B)**, and their phylogenetic affiliation. Significantly different (*t*-test, *P*_*adjust*_ < 0.05) groups are denoted with different letters.

To elucidate if the taxonomic distribution patterns of fungal communities were associated with their potential specific function, we further investigated the elevational patterns of the two predefined functional groups, ectomycorrhizal (ECM) and saprotrophic fungi. The relative abundances of ECM and saprotrophic fungi were summarized by artificially screening sequences that are phylogenetically associated with the previously identified ECM and saprotrophic fungal genera. A total of 41 ECM fungal genera were selected in this study, including 27 Basidiomycotal genera and 14 Ascomycotal genera (Table S1). Within the 41 genera of ECM fungi, 98.03% of the sequences belonged to phylum Basidiomycota. Consequently, the elevational pattern of the relative abundance of ECM fungi was in line with the pattern of Basidiomycota (Figure 3E), with highest abundances at mid-elevation (Figure 6A).

A total of 16 saprotrophic (or potential saprotrophic) fungal genera were selected, including 13 Ascomycotal genera, 2 Basidiomycotal genera, and 1 Zygomycotal genus. Of the total 2,211 selected saprotrophic fungal sequences, 346 sequences were phylogenetically associated with Ascomycota (accounting for 15.65% of all saprotrophic fungal sequences), and 263 sequences associated with Basidiomycota (11.90%). Though only one genus, *Mortierella*, was selected from Zygomycota, a large proportion (74.85%) of the selected saprotrophic fungal sequences were associated with this genus. The relative abundances of saprotrophic fungi in KP-SF forest was much lower than the other vegetation types (Figure 6B), exhibiting a pattern consistent with that of Ascomycota (Figure 3F). The proportions of Ascomycotal saprotrophic fungi were much higher in BL-KP and AT, whereas the Basidiomycotal saprotrophic fungi represented only a small percentage of the total saprotrophic fungi in AT ecosystem.

### Correlations between the relative abundances of dominant taxa and soil properties

**Figure 7:**
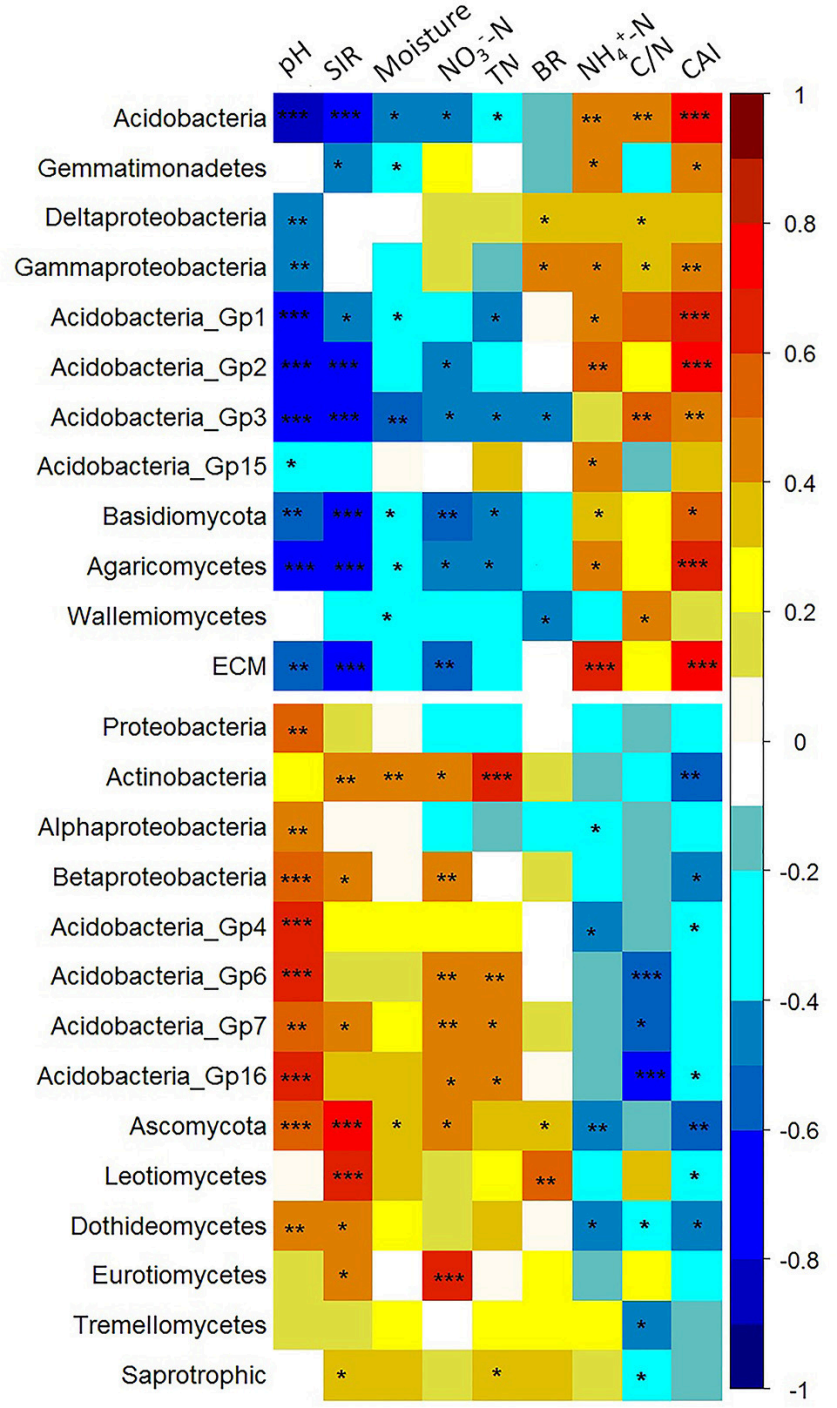
Pearson's correlation coefficients between the relative abundances of specific microbial taxa and the selected soil properties. The correlation coefficients ranging from negative to positive are indicated by color intensity changing from dark blue to red, as illustrated by the figure legend. In general, it was shown that the relative abundances of the so-called “oligotrophic” microbial groups (located at the upper panel) showed positive correlations with ${\text{NH}}_{4}^{+}$-N, C/N and CAI (covered by orange to red color), but negative correlations with TN, ${\text{NO}}_{3}^{-}$-N, moisture content, SIR and pH (covered by cyan and blue color). In contrast, the relative abundances of the “copiotrophic” members (the lower panel) demonstrated the reverse patterns. ^*^0.01 < *P* < 0.05, ^**^0.001 < *P* < 0.01, ^***^P < 0.001.

We performed correlation analysis to examine the relationships between the relative abundances of different bacterial and fungal taxa and selected soil properties. The calculated Pearson correlation coefficients between the relative abundances of specific microbial taxa and the measured soil properties are shown in Table S3, and indicated by color intensity in Figure 7. For the bacterial community, the relative abundances of Acidobacteria and Gemmatimonadetes, classes of Gamma- and Delta-Proteobacteria, and Acidobacteria_Gp1, Gp3, Gp5, Gp15 showed general positive correlations with ${\text{NH}}_{4}^{+}$-N, C/N, and CAI, but negative correlations with TN, ${\text{NO}}_{3}^{-}$-N, moisture content, SIR, and pH. In contrast, the relative abundances of the phylum of Actinobacteria, classes of Betaproteobacteria, and Acidobacteria_Gp4, Gp6, Gp7, and Gp16 showed a reverse pattern, being negatively correlated with ${\text{NH}}_{4}^{+}$-N, C/N, and CAI. The proportion of the overall Proteobacteria was positively correlated with soil pH (*P* < 0.05). As for the fungal community, the relative abundances of Basidiomycota, class of Agaricomycetes and ECM fungi were positively correlated with CAI (*P* < 0.05), but negatively correlated with TN, ${\text{NO}}_{3}^{-}$-N, moisture content, SIR and pH, whereas those of Ascomycota, classes of Leotiomycetes, Dothideomycetes, Eurotiomycetes, and Tremellomycetes, and saprotrophic fungi illustrated an opposite pattern. In summary, it was found that the microbial taxa with higher relative abundances at mid-elevation generally showed positive correlations with ${\text{NH}}_{4}^{+}$-N, C/N, CAI and negative correlations with TN, ${\text{NO}}_{3}^{-}$-N, moisture content, SIR and pH. The redundancy analysis (RDA) based on the relative abundance of selected microbial taxa revealed that the soil microbial biomass, BR and SIR were positively correlated with the copiotrophic microbial taxa (Figure S2). The results of HLM also indicated that pH, TN, SIR, and CAI could explain the variation in the relative abundance of selected taxa (Table S4). This was in line with the Pearson's correlation analysis which showed that the relative abundance of almost all copiotrophic taxa increased with increasing pH and TN. In contrast to the copiotrophic taxa, pH and TN shows negative relationship with oligotrophic taxa (Table S4).

## Discussion

### Ecological classification of oligotrophic and copiotrophic microbial taxa

The mechanisms and theory explaining elevational patterns of soil microbial alpha-diversity have been frequently proposed. For example, soil pH is often suggested as the key factor driving elevational alpha-diversity patterns of soil microbes (Bryant et al., 2008; Singh et al., 2012; Shen et al., 2013; Wang J. T. et al., 2015; Zhang et al., 2015). Moreover, the elevational pattern of bacterial alpha-diversity was also suggested to be influenced by aboveground vegetation diversity (Zhang et al., 2015). The fungal beta-diversity has been proposed to be mainly related to soil organic matter (Zinger et al., 2011). However, the mechanisms driving the distribution pattern of the relative abundance of specific microbial taxa along mountain slopes is still not well-understood. In this study, we described the composition of bacterial and fungal communities along the northern slope of Changbai Mountain and used a oligotrophic-copiotrophic theory to explain the elevational distribution pattern of the relative abundance of dominant soil microbial taxa.

Consistent with our initial hypothesis, different vegetation types across elevational gradients harbored distinct soil bacterial and fungal communities. In particular, the well-defined oligotrophic bacterial phylum, Acidobacteria (Smit et al., 2001; Ramirez et al., 2010, 2012; Fierer et al., 2012; Li et al., 2014, 2016; Pan et al., 2014), had a greater relative abundance in the two dark-coniferous forests at mid-elevation, habitats which were relatively nutrient-poor compared to the other sites. In contrast, Proteobacteria, which is generally considered to be copiotrophic (Fierer et al., 2007; Francioli et al., 2016), did not change in relative abundances across the elevation gradient.

There is an enormous amount of phylogenetic and physiological diversity within each phylum, and it is unlikely that an entire phylum would share common ecological characteristics. Thus, we further examined taxa distributions at finer taxomomic levels within the phyla of Acidobacteria and Proteobacteria. In the phylum of Proteobacteria, it is generally accepted that the Alpha- and Beta-Proteobacteria are copiotrophic members (Fierer et al., 2007; Zhao et al., 2014), because they are usually associated with habitats having relatively low soil C/N ratio, high ${\text{NO}}_{3}^{-}$-N (Nugroho et al., 2005), and enriched nutrients (Leff et al., 2015; Li et al., 2016). The ecological classification of Gammaproteobacteria is less clear (Cho and Giovannoni, 2004; Cleveland et al., 2007; Fierer et al., 2007; Leff et al., 2015). Deltaproteobacteria is typically classified into the oligotrophic group, as revealed by the relatively low abundances in nutrient rich habitats (Banning et al., 2011). Our results are generally consistent with previous ecological classification of proteobacterial classes, with Alpha- and Beta-Proteobacteria having the highest relative abundances at the nutrient rich sites and Gamma- and Delta-having the highest relative abundance in the nutrient-poor dark-coniferous sites.

The present study showed that within the phylum of Acidobacteria, the subgroups of Acidobacteria_Gp1, Gp2, Gp3, Gp15 had a higher relative abundance in the two dark-coniferous forests. In contrast, members of Gp4, Gp6, Gp7, and Gp16 had higher relative abundances in forests with broad leaf trees. Our results are in line with a variety of previous studies. For example, it was documented that Gp1 and Gp3 were more abundant in bulk soil (defined as a low-nutrient habitat) than in the rhizosphere soil (a typical nutrient-rich niche), whereas Gp6 showed the potential predominance in the rhizosphere soil (Nunes da Rocha et al., 2013). A study on forest-to-pasture conversion found that Acidobacteria subgroups Gp1, 3, 5, 9, 11, and 13 were significantly associated with forest soils and were negatively correlated with soil nutrients contents, while subgroups 4, 6, 7, 10, 17, 18, and 25 were associated with pasture soils, and were positively correlated to nutrient availability (Navarrete et al., 2015). There is also genetic evidence supporting our classification of Gp1 and Gp3 subdivisions into oligotrophic categories: Genome analysis of representative Gp1 and Gp3 strains revealed that their genomes encode low-specificity major facilitator super family transporters and high-affinity ABC transporters for sugars, suggesting a suitability to low-nutrient conditions (Ward et al., 2009).

Examining the distributions of other abundant bacterial phyla provided some insight into their trophic categories. For example, the relative abundance of Gemmatimonadetes was much higher in the two dark-coniferous forests, implying oligotrophic tendencies. Gemmatimonadetes has also been proposed as oligotrophic taxa based on other studies, evidenced by a decline in relative abundance under N enrichment (Zhang et al., 2003; Cederlund et al., 2014), and an increase in absolute abundances in dryer soils (DeBruyn et al., 2011). In addition, cultivated Gemmatimonadetes strains grow like oligotrophs, preferring minimal media and exhibiting slow growth rates (DeBruyn et al., 2013). Thus, the relatively low soil moisture and high C/N ratios in the coniferous forests likely contributed to the increased relative abundance of Gemmatimonadetes at those sites.

The ecological category of Actinobacteria has not been clearly defined, with previous studies reporting mixed results in response to nutrient additions (Fierer et al., 2012; Ramirez et al., 2012; Zhao et al., 2014; Leff et al., 2015; Li et al., 2016). Actinobacteria were long believed to behave like fungi, growing slowly and thus behaving like oligotrophs. However, they also play important roles in decomposing the soil organic matter (Dignac et al., 2005), which may imply copiotrophic tendencies. In this study, it was observed that the relative abundance of Actinobacteria was highest in the BL-KP forest, and lower in the two dark-coniferous forests, suggesting a copiotrophic tendency. The inability to definitively classify the entire phyla is likely due the diverse morphological and metabolic repertoires of different Actinobacteria (Embley and Stackebrandt, 1994; Boone and Castenholz, 2001; Garrity and Holt, 2001).

Though the oligotrophic-copiotrophic theory has been intensively discussed within the domain of bacteria, it is less often applied to soil fungal taxa. The Basidiomycota and Ascomycota are two commonly reported soil fungal phyla in forest soils (Frankland, 1998; McGuire et al., 2013). Interestingly, we observed the opposite distribution patterns of these two fungal phyla along the mountain slope. Basidiomycota had the highest relative abundances at the dark forest sites, similar to the oligotrophic bacteria, whereas Ascomycota had the highest relative abundances at the nutrient rich sites, consistent with bacterial copiotrophs. The phenotypic and phylotypic differences between ECM (which are mainly Basidiomycetes) and saprotrophic fungi (which are mainly Ascoymcetes) may support our classification of Basidiomycota and Ascomycota into the oligotrophic and copiotrophic categories, respectively. Overall, mycorrhizal and saprotrophic fungi are likely to be spatially segregated and play fundamentally different roles in the ecosystem. ECM fungi are known to be oligotrophic, and are usually more abundant in deeper soil depths with fragmented litter and humus (Lindahl et al., 2007; McGuire et al., 2013). In contrast, saprotrophic fungi usually exhibit copiotrophic tendencies because they are more efficient in utilizing fresh, energy-rich litter (Lindahl et al., 2007; Crowther et al., 2012) and colonizing nutrient-rich habitats, such as the surface of the forest floor (Lindahl et al., 2007; Lievens et al., 2015). Moreover, previous studies suggested that increasing N supply generally reduced the relative abundances of mycorrhizal fungi (Högberg et al., 2003; Treseder, 2004), such as the Basidiomycetes *Cortinarius* and *Russula* (Lilleskov et al., 2001), but increased the biomass of saprotrophic fungi (Högberg et al., 2003). The different response of ECM and saprotrophic fungi to N enrichment may rely on the opposing nutrient limitations governing C-supply. Specifically, increased N-supply reduces C-supply to plant roots and their mycorrhizal symbionts, while saprotrophic fungi enhance the C-supply due to increased plant-biomass (Högberg et al., 2003). A recent study of genomes of selected mycorrhizal and saprotrophic fungi strains found that ectomycorrhizal fungi have a reduced complement of genes encoding plant cell wall-degrading enzymes, but a rapid genetic turnover in symbiosis-induced genes, compared to their saprotrophic precursors. The convergent losses of ancestral saprotrophic components lower the decomposition ability of ECM fungi (Kohler et al., 2015). Nevertheless, interpretation of ECM fungi data in this study is limited, because ITS sequencing of bulk soil is unlikely to capture the whole profile of ECM fungi (Dickie et al., 2002). Morphological identification is still essential to identify ECM (Smith and Read, 2008), and further ultrastructural or morphological characterization in combination with DNA-based methods would be needed for obtaining a more comprehensive ECM community profile.

The classes within the fungal phylum of Basidiomycota had different distributions, with Agaricomycetes showing an oligotrophic trend and Tremellomycetes demonstrating a copiotrophic tendency. It was suggested that the loss of aggressive ligninolysis in Agaricomycetes has occured in the evolution toward biotrophic ectomycorrhiza (Eastwood et al., 2011), and thus they behave as oligotrophs. Moreover, previous research has shown that *Cortinarius* and *Russula* Agaricomycetes favor lower N habitats, with relative abundances inversely related to nitrification rates (Lilleskov et al., 2002). In contrast, the Tremellomycetes *Cryptococcus* has been characterized as a cellulose degrader (Thongekkaew et al., 2008; Bastias et al., 2009) and orders *Cystofilobasidiales* and *Filobasidiales* have the ability to metabolize easily utilizable nutrients (Liu et al., 2015), indicating copiotrophic traits.

### Vegetation-mediated trophic niche differentiation of soil microbial communities along elevational gradients

An additional goal of this study was to determine which soil factors where most related to taxa distributions. Vegetation types that harbored more copiotrophic microbial taxa, including BL-KP forest, BE forest and tundra, were usually characterized by higher soil moisture, TN, ${\text{NO}}_{3}^{-}$-N, SIR, but lower C/N ratio, ${\text{NH}}_{4}^{+}$-N, and CAI, compared with the two dark-coniferous forests. Thus, relative abundances of copiotrophic microbial taxa were generally correlated with high levels of soil moisture, TN, ${\text{NO}}_{3}^{-}$-N, SIR, whereas the that of oligotrophic members were associated with high levels of C/N ratio, ${\text{NH}}_{4}^{+}$-N, and CAI (Figure 7). These observations suggested that these soil parameters may serve as the indicators for predicting the nutrient status of a given ecosystem, and subsequently, the relative abundance of copiotrophic/oligotrophic taxa.

Soil C/N ratio can reflect the substrate quality for soil microbial growth, and is proposed as a major factor in determining soil microbial community structure (Rousk et al., 2010). Consistent with the current research, a number of previous studies demonstrated that copiotrophic microbial taxa tended to be more abundant in soils with low C/N ratio, and that oligotrophic members preferred habitats with high C/N ratios. For example, Proteobacteria was found to be more abundant in medium-N-addition with relative lower C/N ratio than in low-N-addition with higher C/N ratio (Wang J. et al., 2015). Higher soil C/N ratio was found to be associated with higher proportions of Acidobacteria, but lower relative abundance of Proteobacteria in managed agroecosystems (Wessen et al., 2010). Needle litters generally have higher C/N ratio and are more resistant to degradation than leaf litters (Chiti et al., 2012), and thus, higher soil C/N ratios are frequently reported in coniferous stands compared with broad leaf stands, or with an increasing proportion of conifers in mixed stands (Barbier et al., 2008; Manzoni et al., 2010).

The soil basal respiration and substrate induced respiration (SIR) are commonly used proxies of the overall activity of soil microorganisms. The respiration rate obtained using the SIR method reflects the short term response, and thus generally captures the activity the fastest growing groups (i.e., r strategists or copiotrophs) (Stenstrom et al., 2001). The RDA analysis revealed that microbial biomass, BR and SIR were all positively associated with the relative abundance of copiotrophic taxa. It was also found that the soil carbon availability (CAI) was positively associated with the relative abundance of oligotrophic taxa. Soils dominated by K members (oligotrophic microbes) do not require much labile carbon to grow, and thus, show weak response to glucose addition (low SIR and high CAI value). Consequently, the proportion of oligo- or copio-trophic microbial taxa in a microbial community may play important roles in predicting soil organic carbon (SOC) mineralization process, and the subsequent C balance in the a given ecosystem.

In this study, we found significant correlations between the relative abundances of some specific microbial taxa and soil pH, an edaphic parameter that is frequently used to explain the variations of soil microbial diversity and community compositions (Fierer and Jackson, 2006; Lauber et al., 2008; Ramirez et al., 2010; Shen et al., 2014; Wang J. T. et al., 2015). The correlations between the relative abundances of specific microbial taxa, such as Acidobacteria, Proteobacteria, Bacteroidetes, Ascomycota, and soil pH have been reported by a variety of previous studies (Lauber et al., 2008; Naether et al., 2012; Meng et al., 2013). Soil pH can be considered as a master variable that integrates a number of other soil and site characteristics, so we do not know whether pH itself would directly influence microbial community composition, or is indirectly related through other environmental parameters, such as soil parent material (Barton et al., 1994), the composition of litter falls (Bernhard-Reversat, 1999), fertilization (Johnston et al., 1986) and organic matter content (Dahlgren et al., 1997).

We initially hypothesized that the alpine tundra was an oligotrophic habitat, with low temperature, short growing seasons, and low nutrient availability. In contrast to our hypothesis, the AT site exhibited characteristics of a copiotrophic niche in this experiment, evidenced by higher soil ${\text{NO}}_{3}^{-}$-N and SIR, lower C/N, and higher proportions of potential copiotrophic microbial taxa. High levels of soil available nutrients and the relatively fast C and N cycling processes in the alpine zone has been reported in previous studies (Bowman et al., 1993; Frangi et al., 2005; Shen et al., 2016). Higher labile carbon in tundra soils compared to forest soils were also documented on Shennongjia Mountain, China (Ding et al., 2015), in the Fennoscandian mountains (Sjögersten et al., 2003), and at our study site, Changbai Mountain (Zhang et al., 2013; Tian et al., 2014). A survey of soil microbial functional genes on Changbai Mountain reported an increase in C and N cycling genes at the tree line ecotone, implying the accelerated nutrient cycling in the tundra zone (Shen et al., 2016). Changbai Mountain tundra was shaped by glacial retreat during the Quaternary period, and the plants remaining above 2,000 m are of polar origin, likely deposited by the melting glaciers (Xu et al., 2004). Because of the low temperature and poor litter quality, plant carbon inputs in tundra soil are very slow (Dai et al., 2002; De Deyn et al., 2008), and this slow carbon allocation may provide sustainable available C resources to soil microbes. The high nutrients level in tundra soils may also rely on the high proportion of belowground plant carbon (root: shoot ratio of 6.6) (Jackson et al., 1996), which was mainly contained in the soil surface due to permafrost (Jobbagy and Jackson, 2000; Schenk and Jackson, 2002). Moreover, the relative high silt/clay proportion in tundra soil on Changbai Mountain may further help maintain soil nutrient concentrations (Tian et al., 2014). These higher available nutrients in tundra soils likely explain the higher relative abundances of copiotrophic taxa we observed (Figures 2–4). Indeed, previous research has also reported higher proportions of Proteobacteria (or Alphaproteobacteria) in alpine soils than in soils below the tree line (Shahnavaz et al., 2012).

## Conclusions

The present study revealed that different vegetation types across elevational gradients on the northern slope of Changbai Mountain each harbors distinct soil microbial communities, in terms of both bacteria and fungi composition. In particular, the potential oligotrophic microbial taxa had higher relative abundances in the two dark-coniferous forests at mid-elevation, whereas the forests with more broad leaf trees and alpine tundra had higher relative abundances of copiotrophic taxa. It implied that the elevational patterns of dominant soil microbial taxa along mountain slope were shaped more by vegetation and soil nutrient status. The present study also expands the oligotrophic-copiotrophic theory from dominant bacterial and fungal phyla to the class level, and provided insights into biotic indicators that discriminate different trophic niches. While it is not possible to reliably infer the life strategy of a microbial population based solely on taxonomic catalogs of gene sequences, the oligotrophic-copiotrophic scheme could be used as a theory to synthesize the ever-increasing amount of taxonomic data on soil microbial communities in an ecologically meaningful manner, providing explanations for observed biogeographical patterns.

## Author contributions

HL and XugW designed the research. FY and JY collected soil samples. FY, SY, ZW, XF, and XueW performed the experiments. JY and XugW provided the plant data. FY and HL performed statistical analyses and prepared the draft of the manuscript. YJ and XugW provided intellectual input and advised on the data analyses. JD edited language and advised on the Figures and Tables. All the authors revised the manuscript and approved the final version.

### Conflict of interest statement

The authors declare that the research was conducted in the absence of any commercial or financial relationships that could be construed as a potential conflict of interest.
