# Supplementary material for: Microbial Taxa Distribution Is Associated with Ecological Trophic Cascades along an Elevation Gradient
Source: Front Microbiol. 2017 Oct 27;8:2071. doi: 10.3389/fmicb.2017.02071 (PMC5663944; doi:10.3389/fmicb.2017.02071)
Supplement: Supplementary file 1 [file DataSheet1.DOC]

Supplementary Material

**Microbial taxa distribution is associated with ecological trophic cascades along an elevation gradient**

***Fei Yao^1, 2^, Shan Yang^1, 3^, Zhirui Wang^1, 2^, Xue Wang^1, 2^, Ji Ye^1^, Xugao Wang^1^, Jennifer M. DeBruyn^4^, Xue Feng^1^, Yong Jiang^1^ and Hui Li^1,*^***

**Correspondence**

Hui Li

Tel: +86-24-83970603; Fax: +86-24-83970300;

E-mail: huili@iae.ac.cn

Supplementary Figures and Tables

# Supplementary Figures and Tables

## Supplementary Figures


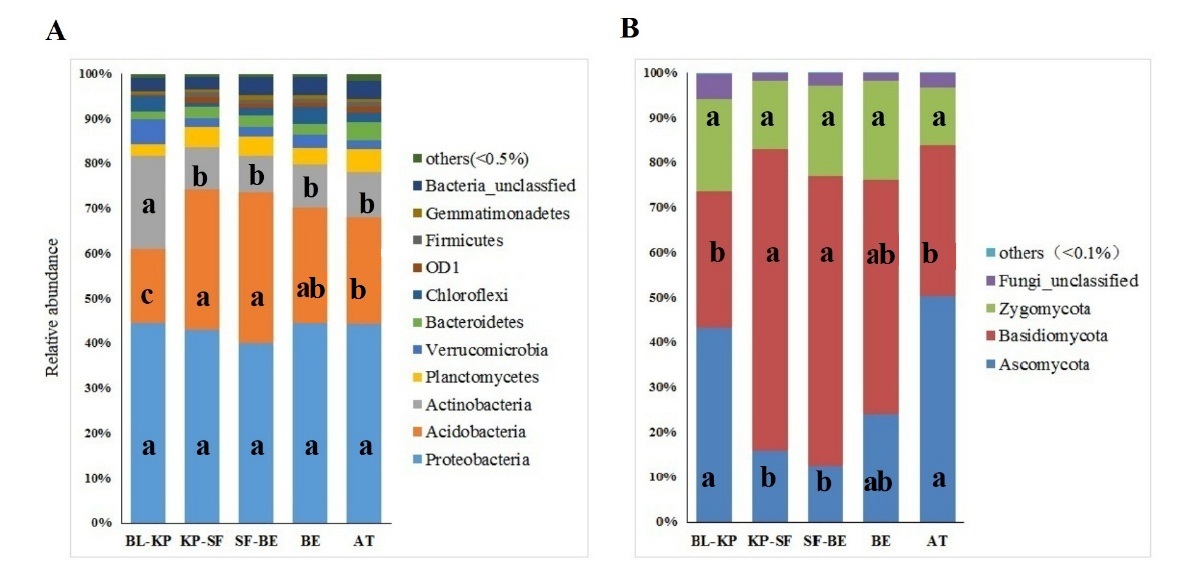


**FIGURE S1 | Mean relative abundances of dominant bacterial (A) and fungal (B) phyla in soils under different vegetation types along elevational gradient.** Significant differences (t test, *P_adjust_* < 0.05) between vegetation types are labeled with different letters. BL-KP, Broad-leaved Korean pine mixed forest; KP-SF, Korean pine-spruce fir forest; SF-BE, Spruce Fir -*Betula ermanii* forest; BE, *Betula ermanii* forest; AT, Alpine tundra.


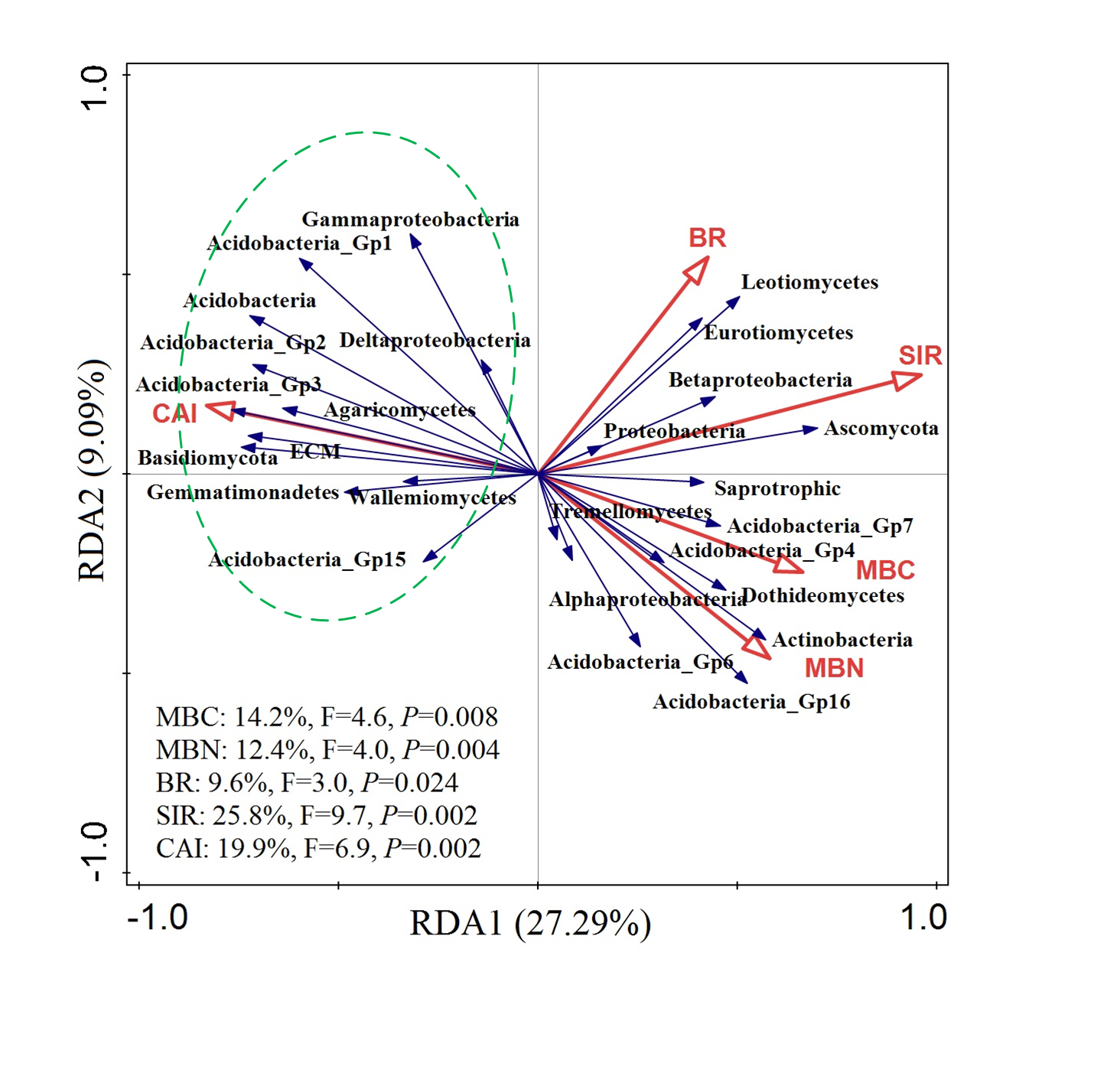


**FIGURE S2 | Correlations of soil microbial biomass, soil respiration parameters and selected microbial taxa as determined by redundancy analysis (RDA). All the parameters showed a significant influence on the microbial community composition, and the percentage of the contribution to the overall variations were shown in the lower left corner.** The dotted circle indicates the potential oligotrophic taxa, and the relative abundance of these taxa showed negative correlations with MBC, MBN, BR, SIR and positive correlations with CAI. The uncircled taxa are the taxa with copiotrophic tendency, showed positive with soil microbial biomass, and soil respiration parameters, but showed negative correlations with CAI. MBC, microbial biomass carbon; MBN, microbial biomass nitrogen; BR, basal respiration; SIR, substrate induced respiration; CAI, carbon availability index

##

## Supplementary Tables

**TABLE S1 | List of selected fungal genus that could be classified as ectomycorrhizal (ECM) or saprotrophic fungi.**

| **ECM** | | | | | **Saprotrophic** | | |
| --- | --- | --- | --- | --- | --- | --- | --- |
| Genus level | Phylum | Genus level | Phylum | Reference | Genus level | Phylum | Reference |
| *Acephala* | *Ascomycota* | *Lactarius* | *Basidiomycota* | Tedersoo et al., 2010 | *Fusarium* | *Ascomycota* | Branco et al., 2013 |
| *Amanita* | *Basidiomycota* | *Lactifluus* | *Basidiomycota* |  | *Mortierella* | *Zygomycota* |  |
| *Amphinema* | *Basidiomycota* | *Meliniomyces* | *Ascomycota* |  | *Cladosporium* | *Ascomycota* |  |
| *Boletus* | *Basidiomycota* | *Membranomyces* | *Basidiomycota* |  | *Cryptococcus* | *Basidiomycota* |  |
| *Byssocorticium* | *Basidiomycota* | *Naucoria* | *Basidiomycota* |  | *Leptosphaeria* | *Ascomycota* |  |
| *Cadophora* | *Ascomycota* | *Pachyphloeus* | *Ascomycota* |  | *Paecilomyces* | *Ascomycota* |  |
| *Cenococcum* | *Ascomycota* | *Porphyrellus* | *Basidiomycota* |  | *Phialophora* | *Ascomycota* |  |
| *Chamonixia* | *Basidiomycota* | *Protoglossum* | *Basidiomycota* |  | *Microdochium* | *Ascomycota* |  |
| *Chloridium* | *Ascomycota* | *Rhizopogon* | *Basidiomycota* |  | *Helotiales* | *Ascomycota* |  |
| *Clavulina* | *Basidiomycota* | *Russula* | *Basidiomycota* |  | *Dothideomycetes* | *Ascomycota* |  |
| *Elaphomyces* | *Ascomycota* | *Suillus* | *Basidiomycota* |  |  |  |  |
| *Genea* | *Ascomycota* | *Tarzetta* | *Ascomycota* |  |  |  |  |
| *Hebeloma* | *Basidiomycota* | *Thelephora* | *Basidiomycota* |  |  |  |  |
| *Humaria* | *Ascomycota* | *Tomentella* | *Basidiomycota* |  |  |  |  |
| *Hydnobolites* | *Ascomycota* | *Tomentellopsis* | *Basidiomycota* |  | *Alternaria* | *Ascomycota* | Frankland, 1998 |
| *Hydnotrya* | *Ascomycota* | *Tricholoma* | *Basidiomycota* |  | *Aureobasidium* | *Ascomycota* |  |
| *Hydnum* | *Basidiomycota* | *Tuber* | *Ascomycota* |  | *Cladosporium* | *Ascomycota* |  |
| *Hygrophorus* | *Basidiomycota* | *Tylospora* | *Basidiomycota* |  | *Phoma* | *Ascomycota* |  |
| *Inocybe* | *Basidiomycota* | *Wilcoxina* | *Ascomycota* |  | *Mycosphaerellaceae* | *Ascomycota* |  |
| *Laccaria* | *Basidiomycota* | *Xerocomus* | *Basidiomycota* |  | *Mycena* | *Basidiomycota* |  |

**TABLE S2 | Pairwise ADONIS test of microbial community similarities between vegetation types based on Bray-Curtis distances.** Shown are the *P* values. BL-KP, Broad-leaved Korean pine mixed forest; KP-SF, Korean pine-spruce fir forest; SF-BE, Spruce Fir -*Betula ermanii* forest; BE, *Betula ermanii* forest; AT, Alpine tundra.

|  |  | **BL-KP** | **KP-SF** | **SF-BE** | **BE** | **AT** |
| --- | --- | --- | --- | --- | --- | --- |
|  |  | *P* | *P* | *P* | *P* |  |
|  | BL-KP |  |  |  |  |  |
| Bacteria    Fungi | KP-SF | 0.002 |  |  |  |  |
|  | SF-BE | 0.003 | 0.001 |  |  |  |
|  | BE | 0.001 | 0.002 | 0.001 |  |  |
|  | AT | 0.001 | 0.001 | 0.001 | 0.001 |  |
|  | KP-SF | 0.001 |  |  |  |  |
|  | SF-BE | 0.001 | 0.001 |  |  |  |
|  | BE | 0.001 | 0.001 | 0.001 |  |  |
|  | AT | 0.001 | 0.001 | 0.001 | 0.001 |  |

**TABLE S3 | Pearson correlation coefficients between specific microbial taxa and soil properties. Bold indicates significant correlations with *P* < 0.05**

|  | pH | SIR | Moisture | NO_3_^-^-N | TN | BR | NH_4_^+^-N | C/N | CAI |
| --- | --- | --- | --- | --- | --- | --- | --- | --- | --- |
| Acidobacteria | **-0.802** | **-0.635** | **-0.436** | **-0.408** | **-0.397** | -0.113 | **0.467** | **0.48** | **0.758** |
| Gemmatimonadetes | -0.027 | **-0.428** | **-0.386** | 0.232 | -0.09 | -0.106 | **0.425** | -0.227 | **0.44** |
| Deltaproteobacteria | **-0.481** | -0.028 | -0.002 | 0.172 | 0.129 | **0.389** | 0.323 | **0.38** | 0.337 |
| Gammaproteobacteria | **-0.466** | -0.083 | -0.215 | 0.11 | -0.151 | **0.443** | **0.421** | **0.387** | **0.495** |
| Acidobacteria_Gp1 | **-0.775** | **-0.453** | **-0.363** | -0.361 | **-0.409** | 0.056 | **0.43** | **0.55** | **0.654** |
| Acidobacteria_Gp2 | **-0.726** | **-0.644** | -0.296 | **-0.434** | -0.275 | -0.065 | **0.546** | 0.212 | **0.783** |
| Acidobacteria_Gp3 | **-0.653** | **-0.632** | **-0.562** | **-0.404** | **-0.446** | **-0.447** | 0.17 | **0.541** | **0.499** |
| Acidobacteria_Gp15 | **-0.37** | -0.315 | 0.056 | -0.067 | 0.312 | -0.022 | **0.435** | -0.167 | 0.331 |
| Basidiomycota | **-0.519** | **-0.667** | **-0.374** | **-0.502** | **-0.446** | -0.306 | **0.38** | 0.29 | **0.541** |
| ECM | **-0.595** | **-0.655** | -0.311 | **-0.522** | -0.291 | -0.073 | **0.629** | 0.297 | **0.763** |
| Proteobacteria | **0.595** | 0.172 | 0.007 | -0.21 | -0.27 | -0.082 | -0.347 | -0.101 | -0.282 |
| Actinobacteria | 0.293 | **0.466** | **0.469** | **0.434** | **0.691** | 0.15 | -0.144 | -0.333 | **-0.512** |
| Alphaproteobacteria | **0.491** | 0.036 | 0.049 | -0.355 | -0.185 | -0.271 | **-0.368** | -0.148 | -0.248 |
| Betaproteobacteria | **0.579** | **0.428** | 0.033 | **0.498** | -0.012 | 0.178 | -0.247 | -0.155 | **-0.459** |
| Acidobacteria_Gp4 | **0.673** | 0.232 | 0.245 | 0.294 | 0.281 | -0.07 | **-0.408** | -0.179 | **-0.362** |
| Acidobacteria_Gp6 | **0.634** | 0.179 | 0.191 | **0.443** | **0.409** | -0.061 | -0.139 | **-0.589** | -0.294 |
| Acidobacteria_Gp7 | **0.516** | **0.412** | 0.25 | **0.497** | **0.455** | 0.183 | -0.131 | **-0.506** | -0.36 |
| Acidobacteria_Gp16 | **0.666** | 0.328 | 0.339 | **0.461** | **0.416** | 0.054 | -0.185 | **-0.659** | **-0.396** |
| Ascomycota | **0.597** | **0.759** | **0.379** | **0.423** | 0.307 | **0.386** | **-0.473** | -0.112 | **-0.595** |
| Saprotrophic | -0.072 | **0.385** | 0.355 | 0.124 | **0.367** | 0.344 | 0.109 | **-0.361** | -0.155 |

**TABLE S4 |** Soil variables that explain the variation in the relative abundance of selected microbial taxa, as estimated by Hierarchical Linear Model (HLM). Models presented here were the best based on lowest Akaike information criterion (AIC) and Bayesian information criterion (BIC). Shown are the coefficient estimates and the values in parentheses are standard deviation. * 0.01< *P* <0.05,** 0.001 < *P* < 0.01, *** *P* < 0.001

|  | pH | SIR | Moisture | NO_3_^-^ -N | TN | BR | NH_4_^+^-N | C/N | CAI |
| --- | --- | --- | --- | --- | --- | --- | --- | --- | --- |
| Acidobacteria | **-11.37(1.92) ***** | 0.79(0.86) | 16.17(13.37) | -0.25(0.87) | **-0.77(0.30)*** | -5.98(4.09) | 0.03(0.03) | 0.19(0.20) | **23.19(10.13)*** |
| Gemmatimonadetes | 0.35(0.19) | 0.05(0.08) | -1.05(1.35) | 0.16(1.21) | 0.03(0.04) | -0.53(0.40) | **7*10^-3^(3*10^-3^)*** | -9*10^-3^(0.01) | 0.76(1.04) |
| Deltaproteobacteria | -0.64(0.38) | -0.26(0.18) | -2.06(2.85) | 0.13(0.18) | 0.04(0.05) | 1.61(0.83) | -6.84*10^-3^(6.88*10^-3^) | 0.03(0.03) | -2.15(2.03) |
| Gammaproteobacteria | -1.45(0.73) | 8.42*10^-3^(0.32) | -3.74(4.82) | 0.18(0.32) | -0.08(0.12) | 1.15(1.53) | -1.74*10-3(0.01) | 0.07(0.09) | 4.57(3.79) |
| Acidobacteria_Gp1 | **-7.71(1.56)***** | 0.48(0.69) | 10.14(10.65) | -0.46(0.70) | **-0.61(0.25)*** | -1.68(3.29) | 0.02(0.03) | 0.22(0.17) | 12.20(8.17) |
| Acidobacteria_Gp2 | **-4.22(0.90)***** | 4.91*10^-3^(0.43) | 6.74(6.96) | -0.32(0.44) | -0.25(0.14) | -0.55(2.01) | 3*10^-4^(0.02) | -0.13(0.06) | 8.03(4.83) |
| Acidobacteria_Gp3 | **-3.23(0.98)**** | **0.11(0.47)** | -1.59(7.58) | -0.13(0.48) | -0.19(0.15) | -2.52(2.19) | 0.01(0.02) | 0.11(0.07) | 1.14(5.23) |
| Acidobacteria_15 | -0.02(0.02) | -0.01(7*10^-3^) | -0.05(0.12) | -3.99*10^-3^(7.93*10^-3^) | 4.84*10^-4^(2.49*10^-3^) | 0.03(0.04) | -1.05*10^-4^(2.99*10^-4^) | 1.37*10^-3^(1.16*10^-3^) | -0.08(0.09) |
| Basidiomycota | -21.56(11.94) | -5.43(5.68) | 107.00(92.56) | -2.28(5.89) | -3.40(1.85) | -4.43(26.76) | 0.28(0.22) | 0.58(0.86) | -46.55(64.22) |
| Agaricomycetes | **-29.92(9.87) **** | -4.12(4.70) | 83.93(76.51) | 0.13(4.87) | **-3.50(1.53)*** | -3.35(22.12) | 0.21(0.18) | -0.09(0.71) | -24.99(53.09) |
| Wallemiomycetes | 2.14(2.03) | 0.64(0.96) | 1.47(15.64) | -0.26(1.00) | -0.05(0.32) | -4.89(4.54) | 0.02(0.04) | 0.30(0.15) | 7.50(10.94) |
| ECM | -0.11(0.21) | 0.08(0.09) | 1.67(1.49) | -0.15(0.09) | -0.05(0.03) | -0.61(0.44) | **0.01(3*10^-3^)**** | 0.02(0.01) | 1.65(1.04) |
| Proteobacteria | **10.42(3.35)**** | 1.85(1.60) | 6.78(25.99) | -2.40(1.65) | -0.48(0.52) | -6.21(7.52) | 0.04(0.06) | 0.16(0.24) | 14.63(18.04) |
| Actinobacteria | -7*10^-3^(0.19) | **-0.22(0.09)*** | -2.61(1.30) | 0.05(0.08) | **0.12(0.03)**** | **1.18(0.43)*** | -3.76*10^-3^(3.32*10^-3^) | -0.04(0.02) | **-3.23(1.04) **** |
| Alphaproteobacteria | **9.57(4.19)*** | 1.87(1.98) | 16.62(31.80) | -3.90(2.04) | -0.24(0.66) | -9.32(9.30) | 0.03(0.08) | 0.05(0.33) | 17.23(22.59) |
| Betaproteobacteria | 3.24(1.38) | -0.41(0.34) | -5.66(5.00) | 0.15(0.35) | 0.28(0.15) | 0.79(1.50) | 0.01(0.01) | -0.01(0.08) | -5.84(3.54) |
| Acidobacteria_Gp4 | **3.03(0.73)***** | -0.05(0.32) | 2.74(5.08) | 0.34(0.33) | **0.24(0.12)*** | -0.22(1.55) | -6*10^-3^(0.01) | 0.07(0.07) | 2.33(3.83) |
| Acidobacteria_Gp6 | **0.54(0.14)***** | 0.01(0.07) | -0.30(1.06) | 0.11(0.07) | **0.05(0.02)*** | -0.25(0.31) | 1.13*10^-3^(2.56*10^-3^) | -0.02(0.01) | 0.44(0.75) |
| Acidobacteria_Gp7 | 0.19(0.09) | 0.03(0.04) | -0.80(0.72) | 0.03(0.05) | 0.03(0.01) | -0.06(0.21) | -2*10^-4^(1.72*10^-3^) | -9.42*10^-3^(6.66*10^-3^) | 0.34(0.50) |
| Acidobacteria_Gp16 | **2.03(7.18)*** | 1.52(1.23) | 3.88(1.85) | -3.18(6.11) | 1.24(0.04) | -6.10(5.22) | -7.02*10^-4^(4.03*10^-3^) | **-1.17(0.02)**** | 2.59(1.22) |
| Ascomycota | 30.03(12.76) | **11.32(3.26)**** | -39.52(52.78) | 1.65(3.22) | 1.49(1.05) | -14.41(15.03) | -0.07(0.13) | 0.48(0.50) | **80.64(35.37) *** |
| Leotiomycetes | 9.11(5.76) | **7.39(2.55)**** | 21.60(39.23) | -2.53(2.57) | 0.45(0.91) | -11.72(12.14) | 0.06(0.10) | 1.05(0.62) | **65.14(30.10)*** |
| Dothideomycetes | 2.70(2.58) | 0.94(1.21) | -33.00(19.32) | -2.04(1.24) | **0.94(0.41)*** | 0.42(5.69) | -0.08(0.05) | -0.36(0.21) | 9.20(13.93) |
| Eurotiomycetes | 1.47(1.76) | 0.25(0.84) | -2.64(13.63) | **3.74(0.87)***** | -0.42(0.27) | 0.75(3.95) | 0.03(0.03) | 0.35(0.13) | -6.41(9.50) |
| Tremellomycetes | 1.71(2.64) | -0.77(1.71) | -15.25(18.90) | -1.29(1.23) | 0.42(0.42) | 5.64(5.69) | 0.02(0.05) | -0.13(0.25) | -13.37(14.07) |
| Saportrophic fungi | -0.69(0.63) | 0.42(0.29) | -0.31(4.63) | -0.20(0.30) | 0.06(0.10) | -1.05(1.38) | -1.05(1.38) | -2.62*10^-3^(0.01) | 4.96(3.38) |
